# Experimental study on the reinforcement performance of unloading-damaged mudstone by microbial grouting

**DOI:** 10.1371/journal.pone.0350062

**Published:** 2026-08-13

**Authors:** Yan Guo, Huafeng Deng, Yongqi Chen, Wenxi Zhu

**Affiliations:** 1 Power China Hubei Electric Engineering Co., Ltd., Wuhan, Hubei, China; 2 College of Civil Engineering & Architecture, China Three Gorges University, Yichang, Hubei, China; 3 Power China Jiangxi Hydropower Engineering Bureau Co., Ltd., Nanchang, Jiangxi, China; Henan Polytechnic University, CHINA

## Abstract

To address the structural deterioration and water-induced swelling of mudstone caused by unloading damage during foundation excavation, MICP and MICP-volcanic ash grouting reinforcement tests were conducted. Mudstone specimens with different unloading damage degrees were prepared through triaxial unloading tests. Combined with physical-mechanical tests and microstructural analyses, the swelling deformation, mechanical properties, and microscopic reinforcement mechanisms of unloading-damaged mudstone under different reinforcement methods were systematically investigated. The results show that unloading damage significantly reduces the strength of mudstone and enhances its swelling sensitivity. Both MICP and MICP-volcanic ash treatments can effectively suppress swelling deformation and improve strength. Specifically, MICP increased the residual strength by 7.41%–13.98% and the peak strength by 2.49%–6.34%, while MICP-volcanic ash increased them by 13.45%–20.61% and 5.36%–9.45%, respectively. Microstructural analysis shows that calcium carbonate mineralization products induced by MICP improve the structural integrity of mudstone by filling pores, sealing cracks, and enhancing interparticle cementation. The introduction of volcanic ash further promotes microbial retention, mineralization deposition, and densification of the pore-fracture structure, resulting in a better reinforcement effect than MICP alone. The results can provide a reference for the green reinforcement and stability improvement of unloading-damaged red-bed mudstone foundations.

## Introduction

In recent years, with the continued expansion of infrastructure construction into red-bed areas, the long-term stability of red-bed mudstone foundations after excavation has attracted increasing attention [1,2]. Under natural conditions, red-bed mudstone generally exhibits a relatively intact primary structure. However, excavation disturbance disrupts the original in-situ stress equilibrium, resulting in significant unloading of the shallow rock mass, which in turn induces crack propagation, structural relaxation, and the development of local damage zones [3,4]. Meanwhile, under the repeated effects of rainfall infiltration and groundwater, the water-induced swelling of clay minerals in mudstone continues to develop, further aggravating structural deterioration and strength degradation of the rock mass, and readily leading to engineering problems such as foundation heave, deformation of retaining structures, and cracking of ancillary facilities [5,6]. Therefore, clarifying the deformation and instability mechanisms of red-bed mudstone under unloading damage and developing corresponding green and efficient reinforcement methods are of great theoretical significance and engineering value for ensuring the long-term service safety of foundations in red-bed areas.

Considerable research has been conducted worldwide on deformation control and stability improvement of red-bed mudstone foundations, mainly through additive modification or grouting reinforcement to enhance the mechanical properties and water stability of mudstone [7]. Previous studies have shown that polyurea can strengthen the bonding between disintegrated mudstone particles and improve their strength stability under wetting-drying cycles [8]; organic acid-modified nano-colloidal silica-aluminate cement composite grouts can reduce the softening zone of mudstone and enhance its overall consolidation performance [9]; hydrophobic modifiers can improve the surface wettability and water absorption characteristics of weathered red-bed mudstone, thereby increasing its shear strength and structural stability [10]; and nano-silica sol grouting has shown good performance in sealing microcracks and improving the seepage resistance of mudstone [11]. Overall, existing studies have made substantial progress in enhancing mudstone strength, suppressing softening, and improving water resistance. However, most currently used modification materials are chemically synthesized or energy-intensive to produce, and may still impose environmental burdens during long-term service, which is not fully consistent with the requirements of green, low-carbon, and environmentally friendly engineering development. Therefore, the exploration of eco-friendly reinforcement technologies for red-bed mudstone foundations has become an important research direction in this field.

Microbially induced calcium carbonate precipitation (MICP), as a rapidly developing biomineralization-based reinforcement technology in recent years, has received extensive attention due to its green, low-pollution, and sustainable characteristics [12,13]. This technique relies on urease-active microorganisms to decompose urea and induce the precipitation of calcium carbonate under suitable chemical conditions, thereby filling, bridging, and cementing pores and fractures within the medium [14,15]. Existing studies have demonstrated that MICP performs well in sand reinforcement, bearing-capacity improvement of foundations, and concrete crack repair [16,17]. In recent years, its application to rock fracture remediation has also made notable progress [18,19]. Some studies have further introduced MICP into mudstone improvement and found that calcium carbonate precipitates can form a certain cementation structure within mudstone pores, thereby improving its water stability and mechanical properties [20]. However, because mudstone is characterized by a complex pore structure, high clay mineral content, and strong water sensitivity, the survival of microorganisms as well as the deposition and spatial distribution of mineralization products within the mudstone are restricted to a certain extent. As a result, the reinforcement effect of conventional MICP still has room for further improvement.

Volcanic ash is a natural porous material with abundant reserves and wide availability, and is characterized by low density, high porosity, and a large specific surface area. It has been widely used in building materials and ecological restoration [21,22]. In engineering materials, volcanic ash is commonly used as a cement admixture and concrete additive in self-compacting concrete and high-performance concrete systems [23]. In the environmental field, it can also be used for coral reef restoration and as a natural filter material and biological carrier for the adsorption of pollutants in water [24,25]. From the perspective of material functionality, the porous structure of volcanic ash is favorable for microbial attachment and growth, while its surface-active sites can provide favorable conditions for calcium carbonate nucleation and deposition. Therefore, volcanic ash has the potential to improve the formation efficiency and spatial distribution of mineralization products during the MICP process. On this basis, introducing volcanic ash into the MICP system to develop a volcanic ash-assisted biomineralization reinforcement method may provide a new approach for the green repair and stability improvement of unloading-damaged red-bed mudstone.

Against this background, this study takes unloading-damaged mudstone obtained from a typical red-bed foundation excavation as the research object. Mudstone specimens with different damage degrees were prepared through triaxial unloading tests, and volcanic ash-assisted MICP grouting reinforcement tests were then carried out. Combined with lateral constrained swelling tests, triaxial compression tests, and scanning electron microscopy (SEM) observations, the deformation characteristics, mechanical response, and microstructural evolution of reinforced unloading-damaged mudstone were systematically analyzed. Furthermore, the reinforcement mechanism of volcanic ash-assisted MICP on unloading-damaged mudstone was revealed from both macroscopic mechanical behavior and microstructural evolution. The results of this study can provide a theoretical basis and technical support for the stability evaluation, green reinforcement design, and engineering treatment of unloading-damaged mudstone foundations in red-bed areas.

## Materials and Methods

### Materials

**Fig 1:**
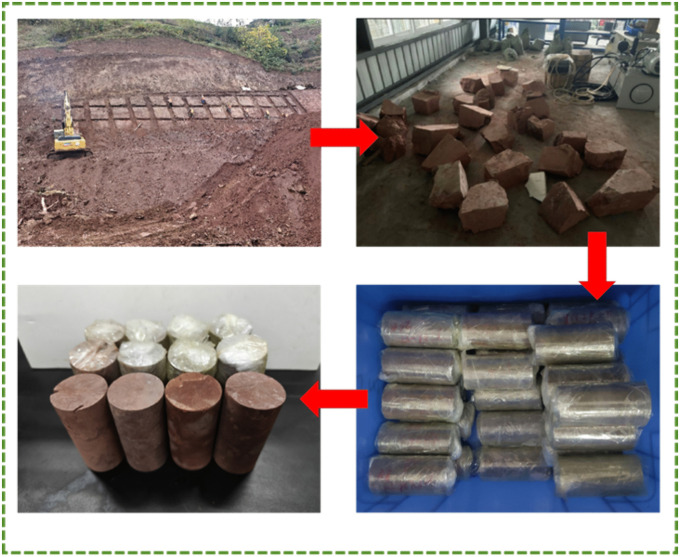
Sampling location of red-bed mudstone and the distributions of P-wave velocity and density.

Red-bed mudstone exposed during foundation excavation at a site in the Badong area was selected as the test material. After field sampling, the rock blocks were transported to the laboratory and processed into standard cylindrical specimens measuring 50 mm in diameter and 100 mm in height in accordance with relevant specifications. Specimens containing visible cracks were discarded. Based on density and P-wave velocity screening, the selected specimens had densities ranging from 2.68 to 2.71 g/cm^3^ and longitudinal wave velocities ranging from 3100 to 3300 m/s, indicating low variability and good homogeneity. The distributions of specimen density and wave velocity are shown in Fig 1.

The mineralizing bacterium used in the MICP process was *Bacillus cereus*, which was independently isolated by the laboratory and is characterized by strong environmental adaptability, high activity, and excellent biomineralization capacity [26]. The bacterial concentration was measured using a UV spectrophotometer, and the OD600 value was determined to be 1.47. A cementation solution containing 1 mol/L calcium chloride and 1 mol/L urea was used to provide the calcium and nitrogen sources required for the MICP reaction.

**Fig 2:**
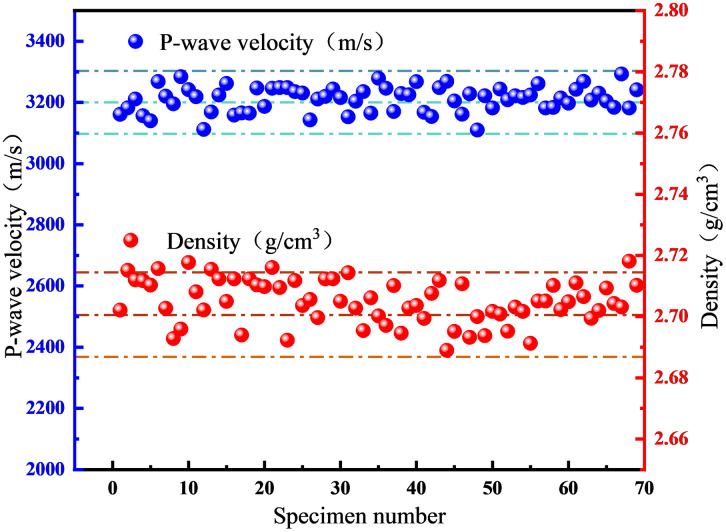
X-ray diffraction pattern of volcanic ash.

The volcanic ash used in the tests was obtained from a material factory in Yichang, Hubei Province, China, and appeared as reddish-brown granular particles. The volcanic ash particles were relatively fine, with particle sizes of less than 2 mm, and had a relatively large specific surface area. To analyze its main mineral composition, X-ray diffraction (XRD) tests were conducted on the volcanic ash, and the results are shown in Fig 2. As shown in the figure, the main crystalline mineral components of the volcanic ash included SiO₂ and Al₂O₃, indicating that it had certain mineral activity and potential conditions to serve as a carrier for microbial attachment and calcium carbonate nucleation.

### Specimen preparation

**Fig 3:**
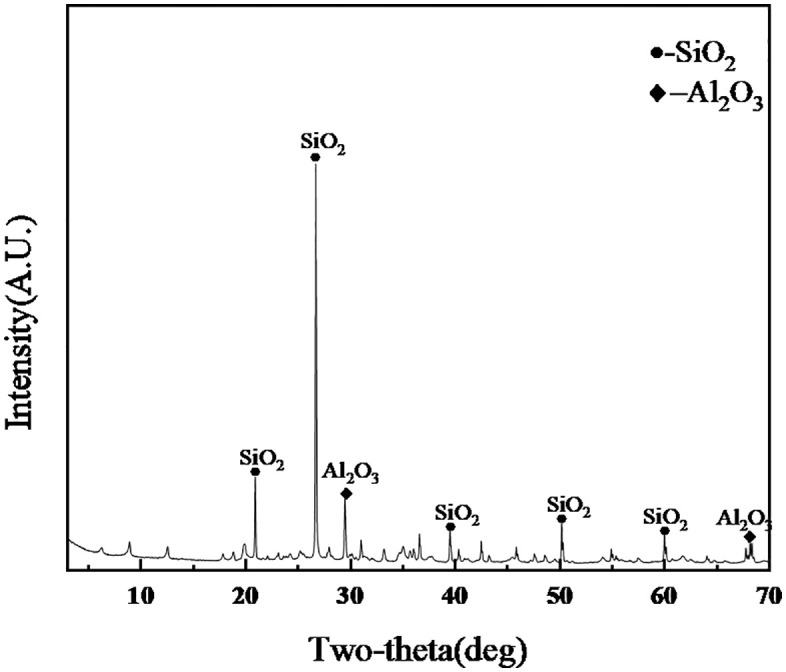
Typical specimen photograph.

To ensure that the bacterial solution and cementation solution could fully infiltrate into the internal fractures of the specimens during the subsequent microbial grouting reinforcement process, a grouting hole with a diameter of 6 mm and a height of 100 mm was pre-drilled along the axial direction of each specimen, as shown in Fig 3. During the subsequent grouting process, the bottom of the grouting hole was first sealed, and then the grouting operation was carried out.

### Experimental scheme

#### Unloading test scheme.

The unloading tests were conducted using an RMT-150C rock mechanics testing system. Conventional triaxial compression tests were first performed at confining pressures of 5, 10, and 15 MPa. During the tests, the confining pressure was loaded to the target value at a rate of 0.05 MPa/s, after which the axial load was applied at a rate of 0.1 kN/s until specimen failure. The stress–strain curves of the mudstone specimens under different confining pressures were obtained, and the peak compressive strength of the specimens under a confining pressure of 5 MPa was determined.

Based on the results of the conventional triaxial compression tests, confining pressure unloading tests were further conducted. In this study, the unloading degree refers to the degree of confining pressure unloading controlled by the stress path, rather than that calculated based on strain. It is defined as follows:


$$\begin{array}{c} \eta =\left[\frac{\sigma_{\text{3}}^{\text{0}}-\sigma_{\text{3}}^{i}}{\sigma_{\text{3}}^{\text{0}}-\sigma_{\text{3}}^{f}}\right]\times \text{1}\text{0}\text{0}\% \end{array}$$

where η is the unloading degree; σ₃⁰ is the initial confining pressure at the unloading point, which was taken as 5 MPa in this study; σ₃ⁱ is the target confining pressure after unloading; and σ₃ᶠ is the confining pressure corresponding to specimen failure under the conventional unloading path. According to the preliminary unloading failure test results, σ₃ᶠ was determined to be 1.6 MPa. Therefore, the target confining pressures of 2.6, 2.3, and 2.0 MPa corresponded to the designed unloading degrees of 70%, 80%, and 90%, respectively. Nine parallel specimens were prepared for each unloading degree, giving a total of 27 specimens, which were used for the subsequent microbial grouting reinforcement tests.

**Fig 4:**
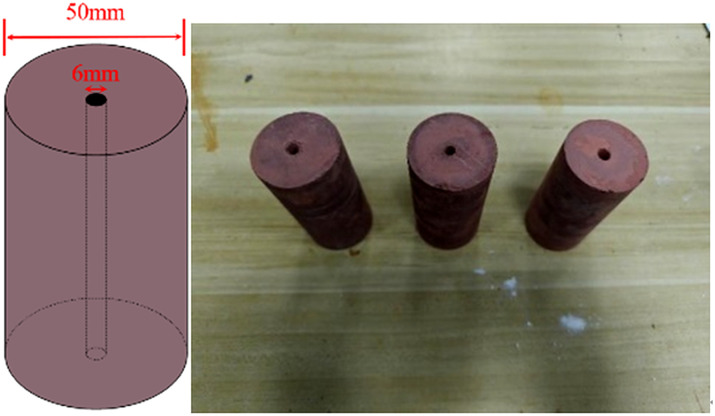
Unloading test scheme (taking the 90% unloading level as an example).

The experimental stress path is shown in Fig 4. During the tests, the axial stress σ₁ and confining pressure σ₃ were first synchronously loaded to 5 MPa. The confining pressure was then kept constant, and the axial load was continuously applied at a rate of 0.1 kN/s. When the axial stress reached 80% of the peak strength under the confining pressure of 5 MPa, this point was defined as the unloading point. Subsequently, the axial load was kept constant, while the confining pressure was unloaded to the preset target values of 2.6, 2.3, or 2.0 MPa at a rate of 0.05 MPa/s. In this way, mudstone specimens with unloading degrees of 70%, 80%, and 90% were prepared. This procedure represents a confining pressure unloading test controlled by the stress path, rather than an unloading process calculated based on strain.

### Reinforcement scheme

Before each grouting cycle, the required bacterial suspension was taken from the conical flask and shaken well. Its bacterial concentration and urease activity were then measured. To ensure the effect of repeated grouting, the OD600 of the bacterial suspension was controlled at about 1.5 before each cycle. The concentration of the cementation solution was 1 mol/L. The volume ratio of bacterial suspension to cementation solution was 1:1.

For the MICP-volcanic ash group, volcanic ash-loaded bacterial suspension was prepared by a pre-loading method. First, dried volcanic ash was weighed at a dosage of 100 g/L and mixed well with the bacterial suspension. The mixture was then placed in a shaking incubator at 30 °C and shaken at 100 r/min for 1 h. This allowed the bacteria to attach to the surface and pores of the volcanic ash particles. The obtained suspension was used as the volcanic ash-loaded bacterial suspension. This treatment helped the porous volcanic ash retain bacteria and provided attachment sites for later calcium carbonate nucleation and precipitation.

Before specimen installation, a thin layer of petroleum jelly was applied evenly to the inner wall of the transparent mold. This was done to reduce friction between the specimen and the mold. Filter paper was also wrapped around the side and bottom surfaces of the specimen. After the specimen was placed in the mold, the mold opening was sealed. The PVC mold was then strengthened with cable ties, and the upper and lower ends were sealed with rubber stoppers.

During grouting, the bottom rubber stopper was opened first. For the MICP group, 20 mL of bacterial suspension was injected into the specimen using a peristaltic pump at a flow rate of 5 mL/min. The specimen was then left to stand for 3 h. After that, 20 mL of cementation solution was injected at the same flow rate. For the MICP–volcanic ash group, 20 mL of volcanic ash-loaded bacterial suspension was injected at the same flow rate. After standing for 3 h, 20 mL of cementation solution was injected. To ensure comparability between the two reinforcement groups, the grouting rate, grouting volume, standing time, and number of cycles were kept the same.

**Fig 5:**
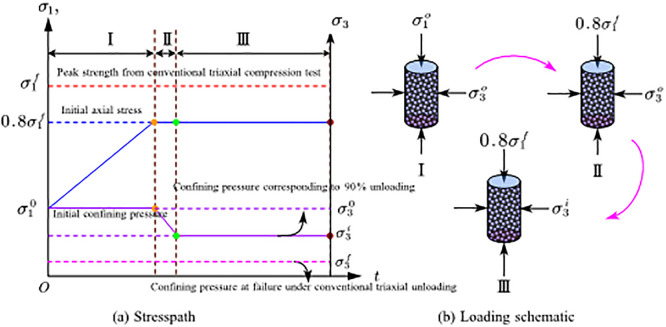
Schematic diagram of the microbial grouting reinforcement device.

**Fig 6:**
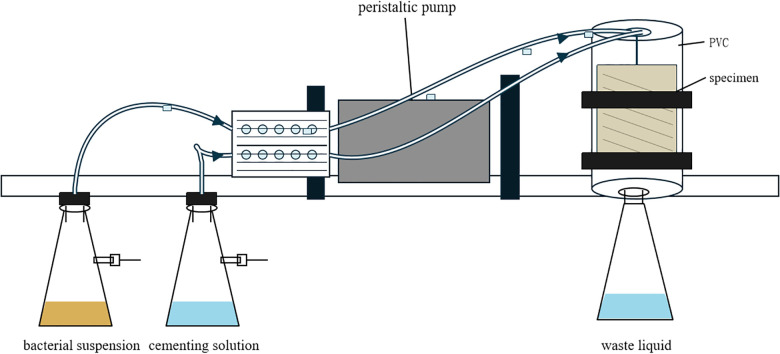
Typical unloading-damaged rock specimens after reinforcement.

After each grouting cycle, the specimens were dried at 40 °C for 12 h. This step was used to reduce the water content of the specimens, promote the stable deposition of mineralization products in pores and fractures, and keep the initial state of the specimens relatively consistent before the next cycle. Since drying may affect the activity of residual microorganisms inside the specimens, fresh bacterial suspension or volcanic ash-loaded bacterial suspension was injected in each cycle. The OD600 and urease activity of the bacterial suspension were also measured before grouting to keep the microbial input conditions as consistent as possible. The above process was defined as one grouting cycle, and four cycles were carried out in total. A schematic diagram of the reinforcement device is shown in Fig 5, and typical reinforced specimens are shown in Fig 6.

As shown in Fig 6, a few microcracks appeared on the surface of the mudstone after unloading damage. After microbial grouting repair, a large amount of calcium carbonate precipitate filled the surface cracks of the unloading-damaged mudstone specimens. This indicates that the repair effect was good.

### Swelling and mechanical properties of reinforced unloading-damaged mudstone

#### Swelling characteristics.

To investigate the effect of MICP reinforcement on the swelling behavior of unloading-damaged mudstone, lateral constrained swelling tests were conducted. During testing, mudstone specimens with different initial unloading damage degrees were placed in a sleeve together with stainless steel porous stones, and a dial indicator mounted on a magnetic stand was used to monitor specimen deformation. Pure water was then slowly injected around the specimen until the water level rose above the upper porous stone, after which the immersion swelling test was performed at room temperature.

**Fig 7:**
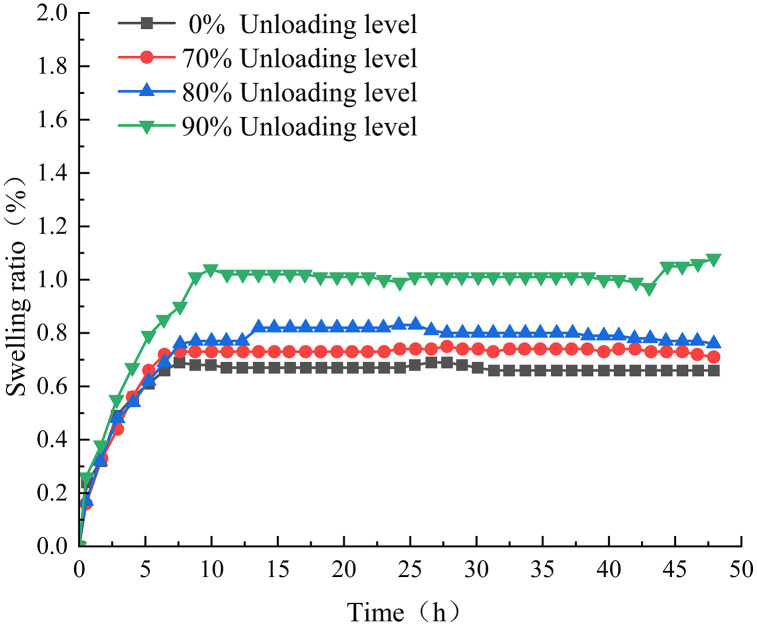
Lateral constrained swelling curves of unloading-damaged mudstone before and after reinforcement Unreinforced specimen.

**Fig 8:**
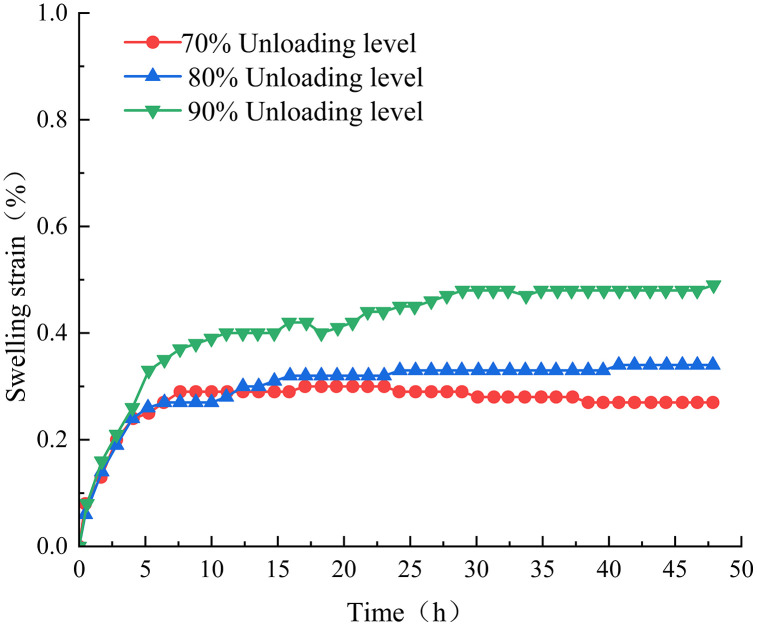
Lateral constrained swelling curves of unloading-damaged mudstone before and after reinforcement Unreinforced specimen MICP-reinforced specimen.

**Fig 9:**
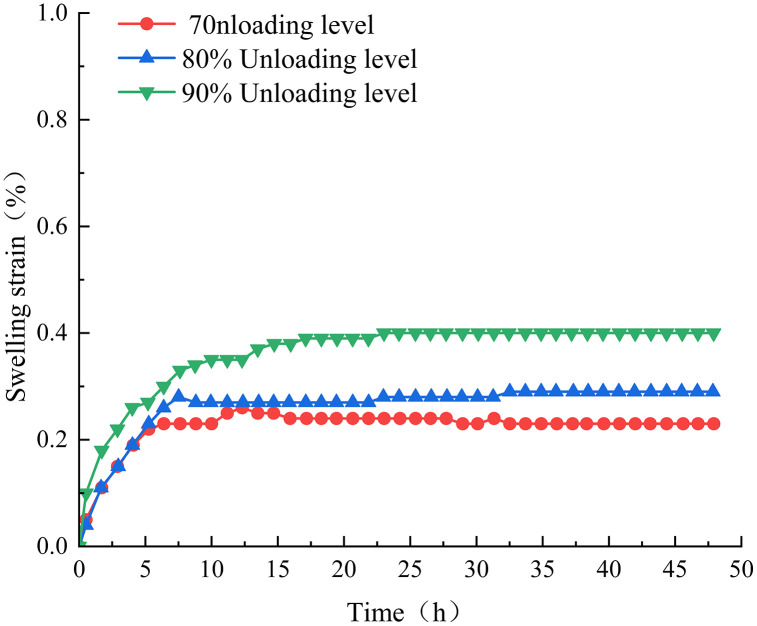
Lateral constrained swelling curves of unloading-damaged mudstone before and after reinforcement Unreinforced specimen MICP-volcanic ash-reinforced specimen.

Specimen deformation was continuously monitored throughout the test. During the initial stage of immersion, readings were recorded every 10 min; after the deformation rate decreased, the recording interval was adjusted to 1 h. When the swelling rate remained below 0.001 mm for three consecutive hours, the swelling deformation was considered to have essentially reached a stable state. The lateral constrained swelling curves of unloading-damaged mudstone before and after reinforcement are shown in Fig 7, Fig 8, Fig 9.

Fig 7-9 shows that all specimens exhibited lateral constrained swelling under continuous water immersion, and the swelling evolution generally followed a three-stage pattern: a rapid growth stage (0–10 h), a slow growth stage (10–30 h), and a stabilization stage (after 30 h). The rapid increase at the initial stage was mainly associated with the swelling of clay minerals and the opening of pre-existing microcracks. Thereafter, the swelling rate gradually decreased as water migration within the specimen’s approached equilibrium, and the swelling strain became nearly stable after 30 h.

For the unreinforced specimens, a pronounced swelling response was observed, and the final swelling strain increased with increasing unloading damage degree, indicating that unloading promoted the development of internal pores and cracks and enhanced the swelling sensitivity of mudstone.

In the MICP-reinforced specimens, a similar stagewise evolution was observed, but the swelling curves were consistently lower than those of the unreinforced specimens under the same unloading condition, and the final swelling strain was significantly reduced. This suggests that MICP reinforcement can effectively suppress the swelling deformation of unloading-damaged mudstone.

A further reduction in swelling strain was obtained after the incorporation of volcanic ash. Compared with the conventional MICP group, the swelling curves of the MICP-volcanic ash-reinforced specimens shifted downward more markedly, and the final swelling strain was further reduced. Overall, both MICP and MICP-volcanic ash reinforcement effectively reduced the lateral constrained swelling strain of unloading-damaged mudstone, with the latter showing a more pronounced inhibition effect.

### Mechanical properties

**Fig 10:**
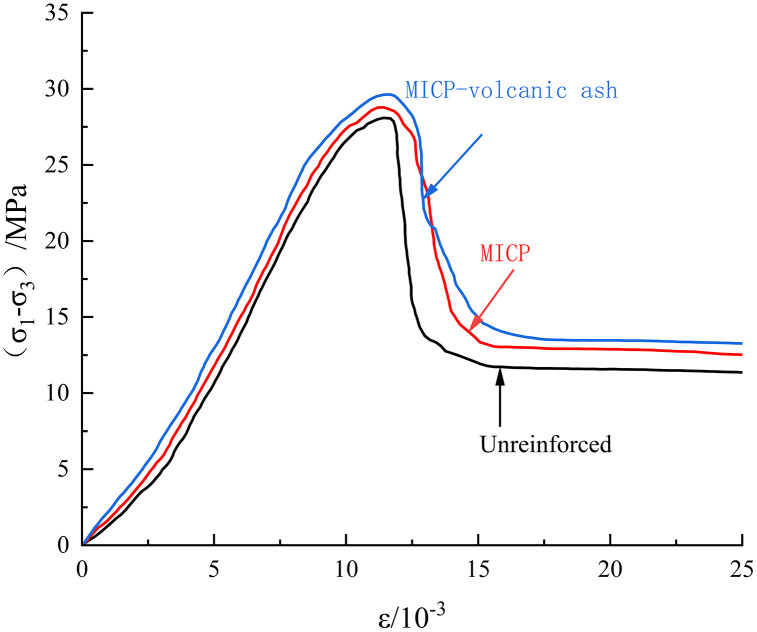
Triaxial compression stress-strain curves of specimens before and after reinforcement Triaxial compression stress-strain curves of specimens with 70% unloading.

**Fig 11:**
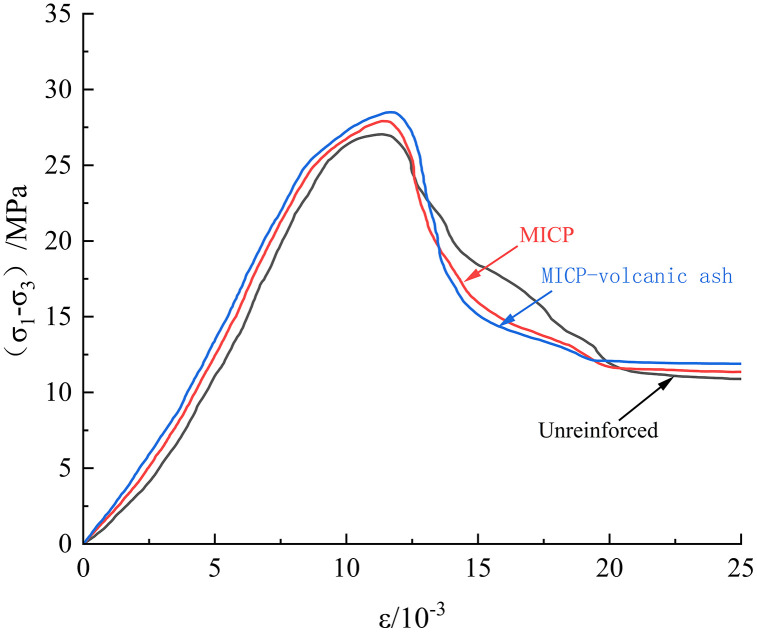
Triaxial compression stress-strain curves of specimens before and after reinforcement Triaxial compression stress-strain curves of specimens with 80% unloading.

**Fig 12:**
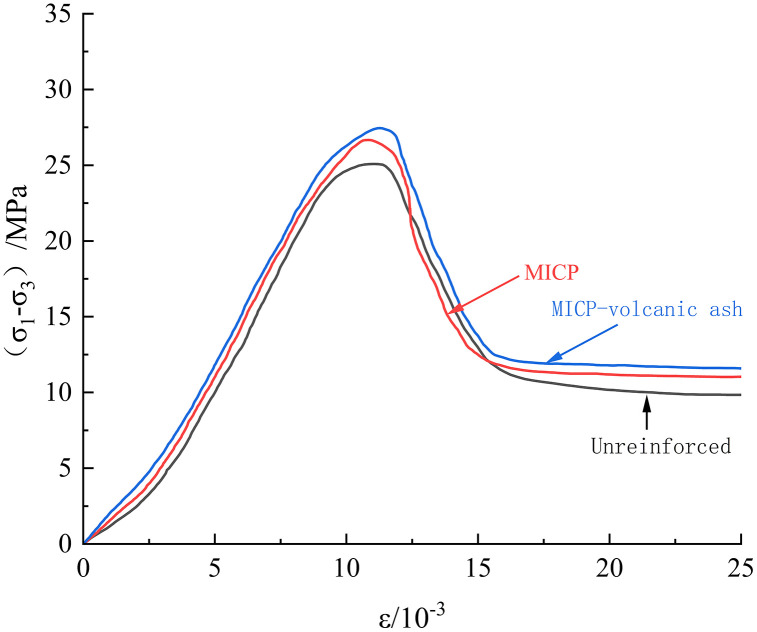
Triaxial compression stress-strain curves of specimens before and after reinforcement Triaxial compression stress-strain curves of specimens with 90% unloading.

To further analyze the mechanical properties of mudstone under unloading damage after MICP reinforcement, triaxial compression tests were carried out on the specimens before and after reinforcement. The corresponding stress-strain curves are shown in Fig 10, Fig 11, Fig 12.

As shown in Fig 10-12, the triaxial compression stress–strain curves of mudstone specimens with different unloading damage degrees showed similar shapes. In general, the curves included the compaction stage, elastic stage, plastic stage, post-peak softening stage, and residual strength stage. As the unloading degree increased, both the peak strength and residual strength of the specimens decreased. This indicates that unloading damage promoted crack development and structural deterioration inside the mudstone, which weakened its bearing capacity.

Under the same unloading condition, the stress–strain curves of the MICP-reinforced specimens were generally higher than those of the unreinforced specimens. Both the peak strength and residual strength increased, and the post-peak softening rate decreased. This shows that MICP reinforcement can effectively improve the mechanical behavior of unloading-damaged mudstone. Further comparison shows that the curves of the MICP–volcanic ash reinforced specimens were generally higher than those of the ordinary MICP-reinforced specimens. Their peak strength and residual strength were further improved, and their post-peak residual bearing capacity was also stronger. This indicates that the addition of volcanic ash further enhanced the filling and cementation effect of MICP, and improved the overall compactness and structural stability of the specimens.

**Fig 13:**
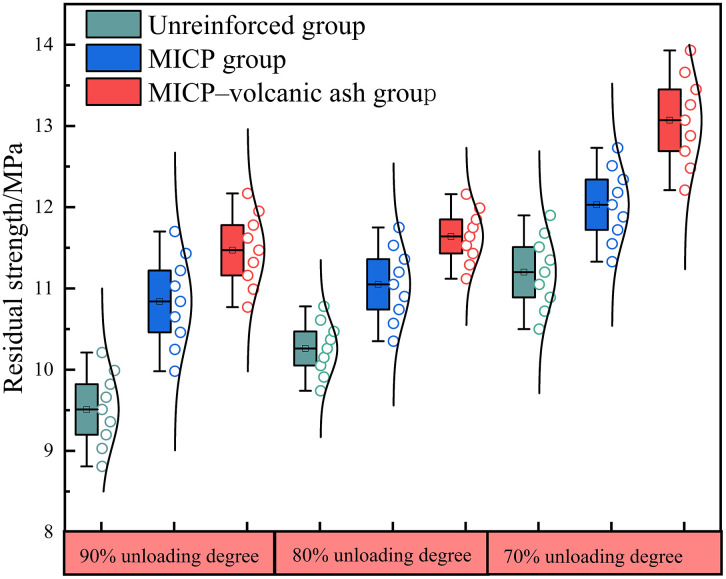
Triaxial compression stress-strain curves of specimens before and after reinforcement Residual strength.

**Fig 14:**
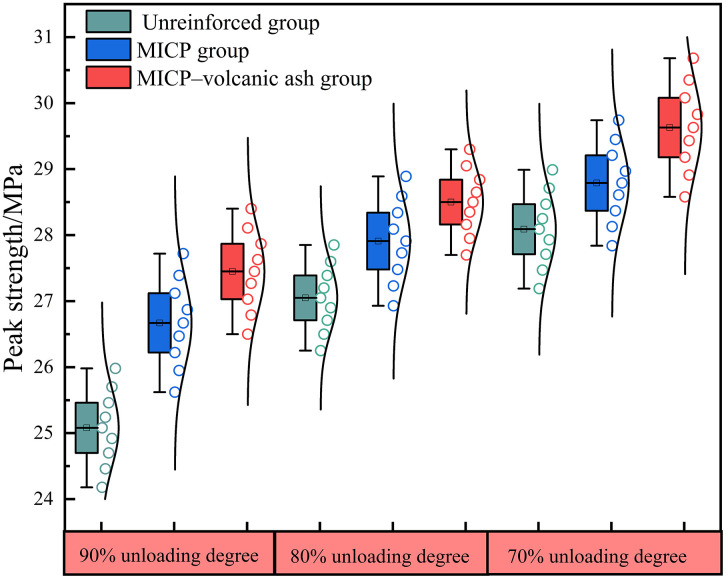
Triaxial compression stress-strain curves of specimens before and after reinforcement Peak strength.

Overall, both MICP and MICP–volcanic ash reinforcement can effectively improve the mechanical properties of unloading-damaged mudstone. The MICP–volcanic ash method showed a better reinforcement effect. To further quantify the strength changes of mudstone before and after reinforcement, the peak strength and residual strength of each group were statistically analyzed. The results are shown in Fig 13, Fig 14 and Table 1.

**Table 1:** Statistical results of compressive strengths of specimens before and after reinforcement.

Unloading damage degree	Treatment method	Residual strength/MPa	CV of residual strength/%	Increase rate of residual strength/%	Peak strength/MPa	CV of peak strength/%	Increase rate of peak strength/%
70%	Unreinforced group	11.20	4.09	–	28.09	2.08	–
70%	MICP group	12.03	3.81	7.41	28.79	2.16	2.49
70%	MICP–volcanic ash group	13.07	4.31	16.69	29.63	2.30	5.48
80%	Unreinforced group	10.26	3.27	–	27.05	1.92	–
80%	MICP group	11.05	4.14	7.69	27.91	2.29	3.18
80%	MICP–volcanic ash group	11.64	2.88	13.45	28.50	1.82	5.36
90%	Unreinforced group	9.51	4.82	–	25.08	2.33	–
90%	MICP group	10.84	5.19	13.98	26.67	2.56	6.34
90%	MICP–volcanic ash group	11.47	3.99	20.61	27.45	2.27	9.45

As shown in Fig 13−14 and Table 1, the residual strength and peak strength of the specimens in different groups showed some discreteness. The CV of residual strength was 2.88%−5.19%, and the CV of peak strength was 1.82%−2.56%. This indicates that the natural heterogeneity of mudstone, differences in unloading damage, and uneven distribution of mineralization products during grouting had some influence on the strength results. However, the overall trend was clear. Under the same unloading damage degree, the residual strength and peak strength of the MICP group and the MICP–volcanic ash group were both higher than those of the unreinforced group. The increase was more obvious in the MICP-volcanic ash group. This shows that the strength improvement after reinforcement was still consistent when specimen discreteness was considered.

For the unreinforced specimens, as the unloading damage degree increased from 70% to 90%, the peak strength decreased from 28.09 MPa to 25.08 MPa, and the residual strength decreased from 11.20 MPa to 9.51 MPa. This indicates that greater unloading damage promoted the development of cracks and pores inside the mudstone. As a result, the bearing capacity and post-peak stability of the specimens were weakened. After MICP reinforcement, the residual strength increased by 7.41%−13.98%, and the peak strength increased by 2.49%−6.34%. After MICP–volcanic ash reinforcement, the residual strength increased by 13.45%−20.61%, and the peak strength increased by 5.36%–9.45%. These results show that both reinforcement methods improved the mechanical properties of unloading-damaged mudstone. The MICP–volcanic ash group showed a better improvement, especially in residual strength.

This was mainly because the calcium carbonate precipitates induced by MICP could fill pores, seal cracks, and strengthen the cementation between particles. This improved the structural integrity of the specimens. In addition, the porous structure and large specific surface area of volcanic ash helped bacterial attachment, retention, and calcium carbonate nucleation. This further promoted the filling and cementation of mineralization products in the pores and cracks of mudstone. Therefore, the MICP-volcanic ash group showed a better strength improvement effect.

### Microstructure and reinforcement mechanism

The swelling behavior and mechanical properties of mudstone with different degrees of unloading damage after MICP and MICP-volcanic ash grouting reinforcement were analyzed in the previous tests. To further reveal the reinforcement mechanism, this section analyzes the microstructural evolution of mudstone before and after reinforcement. The analysis is based on SEM observations, XRD phase analysis, and statistical results of surface porosity and fractal dimension obtained from SEM images. The reinforcement mechanisms of MICP and MICP-volcanic ash for unloading-damaged mudstone are also discussed.

### Microstructure and phase analysis

**Fig 15:**
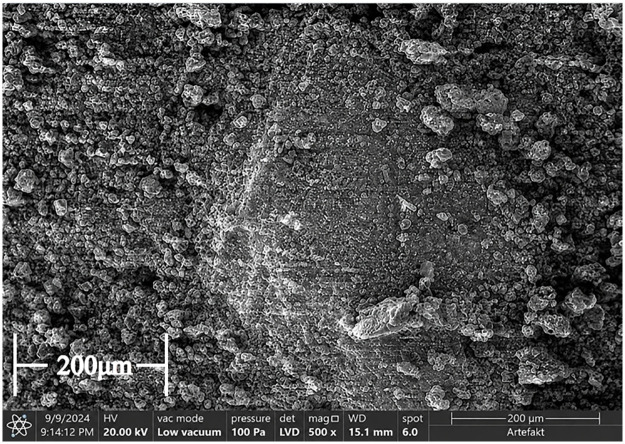
SEM image and binarized image of the unreinforced mudstone specimen SEM image.

**Fig 16:**
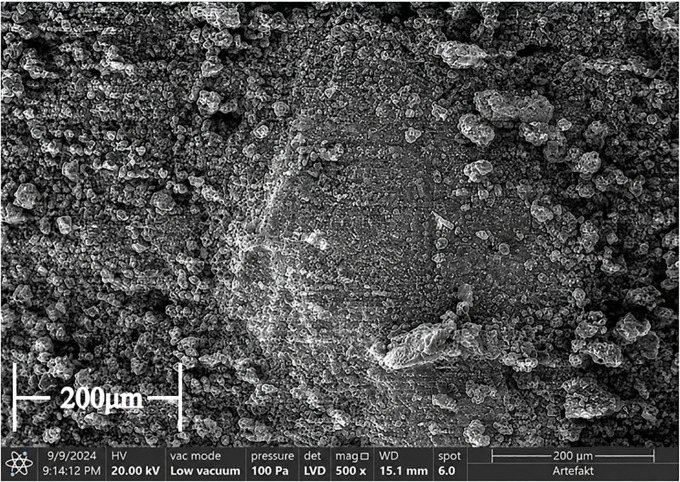
SEM image and binarized image of the unreinforced mudstone specimen Binarized image.

**Fig 17:**
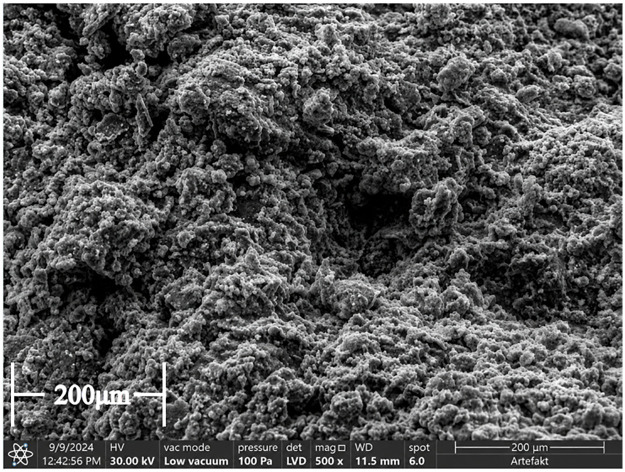
SEM image and binarized image of the mudstone specimen after MICP reinforcement SEM image.

**Fig 18:**
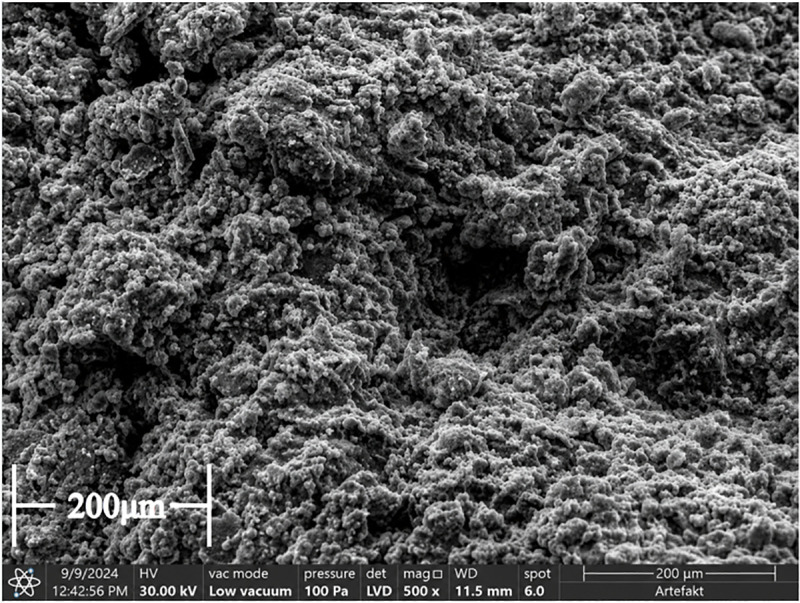
SEM image and binarized image of the mudstone specimen after MICP reinforcement Binarized image.

**Fig 19:**
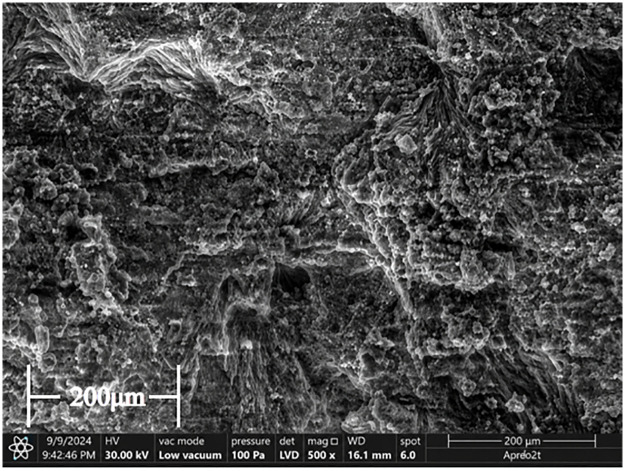
SEM image and binarized image of the mudstone specimen after MICP-volcanic ash reinforcement SEM image.

**Fig 20:**
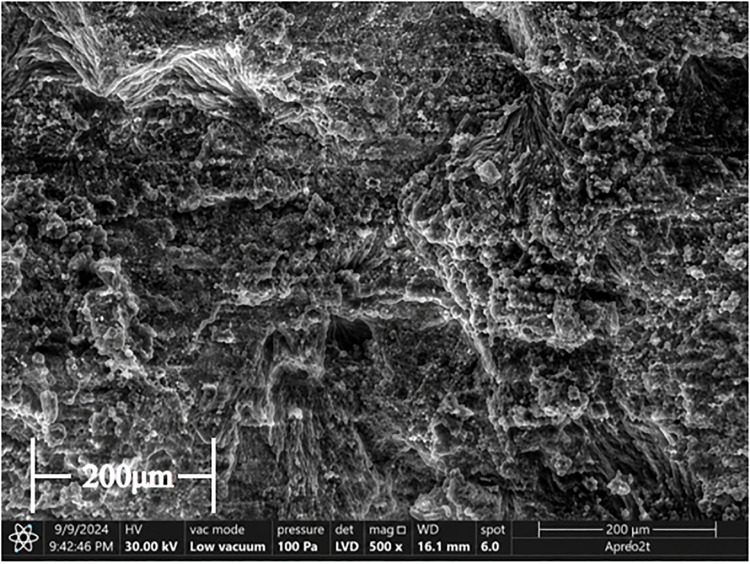
SEM image and binarized image of the mudstone specimen after MICP-volcanic ash reinforcement Binarized image.

**Fig 21:**
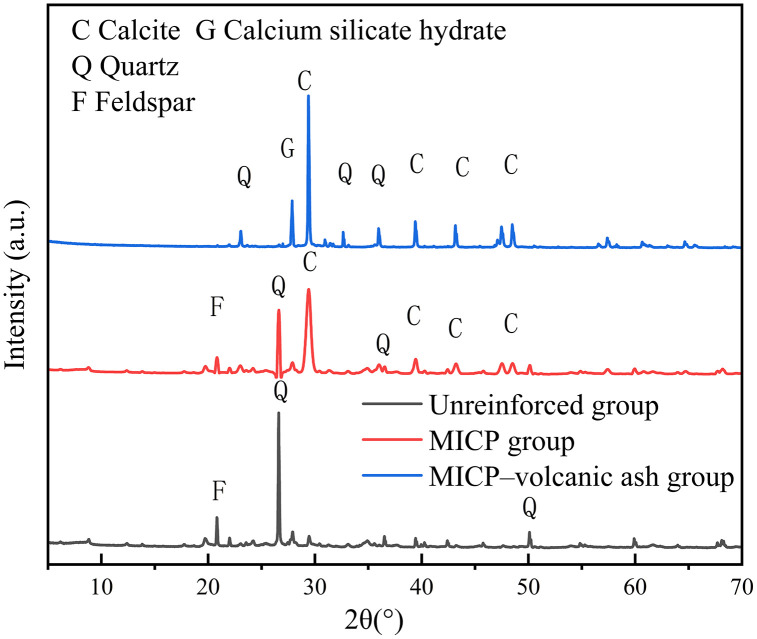
XRD phase analysis results of unloading-damaged mudstone before and after reinforcement.

**Fig 22:**
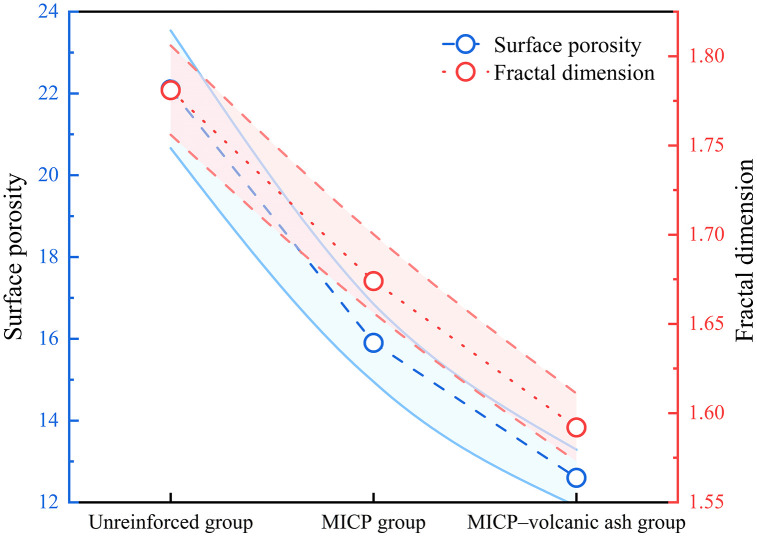
Variations in surface porosity and fractal dimension of unloading-damaged mudstone before and after reinforcement.

Changes in macroscopic mechanical properties are usually closely related to the evolution of the rock microstructure. To further analyze the effect of MICP reinforcement on the microstructure of mudstone, SEM observations were carried out on mudstone specimens before and after reinforcement. The SEM images were also processed by binarization. The SEM images and binarized results of the unreinforced group, MICP group, and MICP–volcanic ash group are shown in Fig 15, Fig 16, Fig 17, Fig 18, Fig 19, Fig 20. The XRD phase analysis results of the specimens before and after reinforcement are shown in Fig 21. The changes in surface porosity and fractal dimension calculated from the SEM images are shown in Fig 22.

As shown in Figs 15–22

(1) The surface structure of the unreinforced mudstone specimen was relatively loose. Some micropores, microcracks, and scattered mineral particles could be observed. The particles were mainly in primary contact, and the cementation between them was weak. This indicates that many pore and crack channels still existed in the mudstone after unloading damage. These channels could provide paths for water migration and conditions for clay minerals to absorb water and swell.

After MICP reinforcement, many granular and clustered mineral deposits appeared on the specimen surface and between particles. Some deposits attached to the pores and cracks and formed bridging and cementation structures between particles. As a result, the original pores and cracks were partly filled and blocked.

For the MICP–volcanic ash reinforced specimen, the deposited structure on the surface was more continuous and denser. The filling degree of pores and cracks was further improved. This shows that the porous structure and large specific surface area of volcanic ash helped bacterial retention and calcium carbonate nucleation. Therefore, the filling and cementation effects of mineralization products in pores and cracks were enhanced.

(2) The XRD results further show that quartz, feldspar, and other original mineral peaks were mainly identified in the unreinforced mudstone specimen. After MICP reinforcement, the diffraction peaks related to calcium carbonate minerals, such as calcite, became stronger. This indicates that the microbial-induced mineralization process promoted the formation of calcium carbonate precipitates.

Compared with the ordinary MICP group, the calcium carbonate-related peaks in the MICP–volcanic ash group were more obvious. This shows that the addition of volcanic ash helped promote the deposition of mineralization products. In addition, a small amount of cementitious products may have formed in the MICP–volcanic ash group. These products may have worked together with calcium carbonate precipitates to form a composite cementation structure. However, EDS point scanning or elemental mapping was not carried out in this study. Therefore, the specific composition of these cementitious products needs to be further verified by micro-area elemental analysis.

(3) The quantitative image analysis results show that the surface porosity and fractal dimension of the unreinforced group were 22.1% and 1.78, respectively. After MICP reinforcement, the surface porosity decreased to 15.9%, and the fractal dimension decreased to 1.66. After MICP–volcanic ash reinforcement, the surface porosity further decreased to 12.6%, and the fractal dimension decreased to 1.59.

Compared with the unreinforced group, the surface porosity of the MICP group and the MICP–volcanic ash group decreased by 28.1% and 43.0%, respectively. This indicates that both reinforcement methods effectively reduced pore development. The improvement was more obvious in the MICP–volcanic ash group. The decrease in fractal dimension shows that the complexity of the pore structure was reduced, and the connectivity of pores and cracks decreased. Therefore, the microstructure of the specimens became denser and more stable.

In summary, MICP mainly filled pores, blocked cracks, and formed particle bridging through calcium carbonate precipitation. The addition of volcanic ash further enhanced bacterial retention, mineralization deposition, and composite cementation. This made the pore and crack structure of mudstone denser. The SEM observations, XRD phase analysis, surface porosity results, and fractal dimension results support each other. They show that both MICP and MICP–volcanic ash reinforcement can inhibit swelling deformation and improve mechanical properties by improving the microstructure of mudstone. The MICP–volcanic ash method showed a better reinforcement effect.

### Mechanism analysis of microbial grouting reinforcement for unloading-damaged mudstone

**Fig 23:**
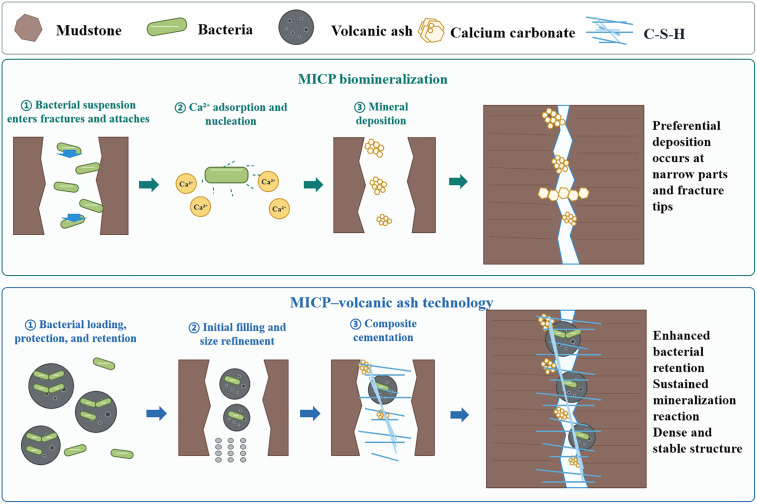
Schematic diagram of the microbial grouting reinforcement mechanism of mudstone.

Based on the above experimental results, the reinforcement effect of MICP on unloading-damaged mudstone is mainly reflected in the following two aspects. The grouting reinforcement mechanisms are illustrated in Fig 23

As can be seen from Fig 23:

(1) During the MICP grouting process, urease-active microorganisms catalyze the hydrolysis of urea to generate carbonate ions, which then induce the precipitation of calcium carbonate in the presence of a calcium source. Because the surfaces of bacterial cells and extracellular polymers contain negatively charged functional groups such as hydroxyl, amino, amide, and carboxyl groups, the bacteria can adsorb Ca^2+^ and serve as nucleation sites for calcium carbonate crystals [27]. As the mineralization reaction proceeds, the generated calcium carbonate precipitates gradually attach to the surfaces of mudstone particles and deposit within pores, cracks, and seepage channels. Through filling, bridging, and cementation, these precipitates enhance interparticle connections and improve the structural integrity and bearing capacity of the specimens.
(2) Furthermore, calcium carbonate precipitates preferentially form and gradually accumulate in narrow crack zones, crack tips, and local seepage-concentrated areas, causing the original unloading-induced cracks to be continuously filled, refined, and sealed [28]. At the same time, the precipitates exhibit a certain degree of adsorption and bonding capacity toward mudstone particles and locally loose cementitious materials, thereby promoting the recommendation of crack surfaces, reducing crack connectivity and permeability, and ultimately realizing the repair and reconstruction of the internal structure of unloading-damaged mudstone. As a result, internal water migration pathways in the mudstone are inhibited, and the water-absorption swelling of clay minerals as well as the associated structural softening effects are correspondingly weakened. This is also the main reason why MICP can suppress the swelling deformation of mudstone and improve its mechanical properties.
(3) Compared with conventional MICP grouting, MICP-volcanic ash grouting further introduces the physical filling effect and chemical enhancement effect of volcanic ash on the basis of biomineralization, and its mechanism is illustrated in Fig 13. Volcanic ash possesses a well-developed pore structure, a large specific surface area, and strong adsorption capacity. During the preparation of volcanic ash-loaded bacterial solution, it can adsorb and enrich a large number of microorganisms, thereby providing an attachment and protective carrier for bacteria, improving their retention capacity and survival time within cracks, promoting the continuous progress of the mineralization reaction, and increasing the yield of calcium carbonate precipitates. Meanwhile, volcanic ash particles themselves can also act as fine filling materials and enter relatively large pores and cracks within the mudstone, providing initial filling and scale refinement of the crack space and creating favorable conditions for the subsequent accumulation of calcium carbonate precipitates. In addition, under alkaline conditions, the active components in volcanic ash may undergo pozzolanic reactions to generate a small amount of C-S-H gel. This gel interweaves with calcium carbonate precipitates to form a composite cementation system, thereby further enhancing the structural stability of the crack zone.
(4) In summary, MICP mainly improves the internal structure of unloading-damaged mudstone through calcium carbonate precipitation, which fills pores, seals cracks, and cements particles. The introduction of volcanic ash further strengthens bacterial retention, precipitate accumulation, and composite cementation effects, enabling the reinforced mudstone to form a denser and more stable microstructure, and ultimately achieving more effective suppression of swelling deformation and further improvement in mechanical properties.

## Discussion

This study confirmed the feasibility of using volcanic ash–MICP to reinforce unloading-damaged mudstone. The porous structure of volcanic ash provides sites for bacterial attachment and calcium carbonate nucleation. Together with MICP mineralization products, it helps fill pores, block cracks, and cement particles. Thus, it improves the resistance of mudstone to swelling deformation and strength deterioration.

However, volcanic ash may also improve the mudstone structure through physical filling and potential reactive effects. Therefore, the reinforcement effect of the MICP–volcanic ash group should be regarded as the combined result of volcanic ash and MICP mineralization. Their individual contributions need further quantitative evaluation through control tests.

In this study, specimens were dried at 40 °C for 12 h after each grouting cycle. This step reduced water content, promoted stable mineral deposition, and provided similar initial conditions for the next cycle. Fresh bacterial suspension or volcanic ash-loaded bacterial suspension was injected in each cycle, and OD600 and urease activity were controlled before grouting. However, the survival rate and urease activity of residual bacteria after drying were not measured. Thus, the effect of drying on later mineralization remains uncertain. Future studies should examine bacterial survival, urease activity, and calcium carbonate production under different drying temperatures and times.

Further research should focus on control tests, process optimization, and field verification. A volcanic ash-only group should be added to distinguish the effects of physical filling, nucleation promotion, and MICP mineralization. Different volcanic ash dosages, particle sizes, and slurry concentrations should also be tested.

For field application, the injectability and diffusion of bacterial suspension, cementation solution, and volcanic ash particles should be studied under different grouting pressures, borehole spacings, grouting volumes, and cycle numbers. In the laboratory, a 6 mm central grouting hole was used to improve slurry penetration. In the field, however, fracture connectivity and seepage paths are more uneven. For low-permeability or weakly fractured mudstone, pre-wetting, local pretreatment, and staged low-pressure cyclic grouting may improve slurry diffusion and reduce the risk of new crack growth.

Finally, field tests are needed to verify the reinforcement effect, long-term durability, and environmental adaptability of volcanic ash-MICP under real engineering conditions. This would support its application in the green reinforcement of red-bed mudstone foundations.

## Conclusions

In this study, MICP and MICP–volcanic ash grouting tests were carried out on unloading-damaged mudstone. Lateral confined swelling tests, triaxial compression tests, SEM observations, XRD phase analysis, and quantitative image analysis were used to study the swelling behavior, mechanical response, and reinforcement mechanism of the specimens. The main conclusions are as follows:

(1) Unloading changed the pore and crack structure of mudstone and caused deterioration in mechanical properties and water stability. As the unloading degree increased from 70% to 90%, cracks and pores developed gradually. The peak strength and residual strength decreased, while swelling deformation under water immersion increased. This shows that unloading damage is an important factor causing strength reduction and higher swelling sensitivity of mudstone.
(2) Both MICP and MICP-volcanic ash grouting effectively reduced the swelling deformation of unloading-damaged mudstone and improved its peak and residual strengths. Compared with the unreinforced specimens, the residual strength of the MICP-reinforced specimens increased by 7.41%−13.98%, and the peak strength increased by 2.49%−6.34%. For the MICP-volcanic ash reinforced specimens, the residual strength increased by 13.45%−20.61%, and the peak strength increased by 5.36%−9.45%. Although some discreteness existed among the specimens, the strength improvement after reinforcement was consistent. The MICP-volcanic ash group showed the best reinforcement effect.
(3) SEM observations, XRD phase analysis, and quantitative image analysis showed that calcium carbonate precipitates formed during MICP could fill pores, block cracks, bridge particles, and improve cementation. These effects improved particle contact, structural compactness, and overall stability. After MICP and MICP–volcanic ash reinforcement, both surface porosity and fractal dimension decreased. This indicates that the reinforcement reduced pore development and the complexity of the pore-crack structure. The improvement was more obvious in the MICP–volcanic ash group.
(4) The reinforcement of unloading-damaged mudstone by MICP is mainly a crack repair and structural reconstruction process based on calcium carbonate mineralization. The addition of volcanic ash further promoted bacterial retention, mineral deposition, and pore-crack filling. It may also form composite cementation together with mineralization products. These effects helped reduce water migration and water-induced swelling softening, and improved both deformation control and mechanical properties. However, the separate contributions of volcanic ash alone and the combined MICP-volcanic ash effect still need to be quantified by further control tests.
