# Supplementary material for: Experimental study on the reinforcement performance of unloading-damaged mudstone by microbial grouting
Source: PLoS One. 2026 Aug 13;21(8):e0350062. doi: 10.1371/journal.pone.0350062 (PMC13472361; doi:10.1371/journal.pone.0350062)
Supplement: Supplementary file 1 [file pone.0350062.s001.docx]

**Manuscript ID: PONE-D-26-22352**

Comments to the Authors,

The authors carried out an experimental investigation on the reinforcement performance of unloading-damaged mudstone through microbial grouting. This paper holds a certain degree of research significance. Nevertheless, the innovation and research methods are mediocre. Consequently, I have pinpointed several aspects that necessitate revision prior to its potential final acceptance.

specific comments：

Section numbering is duplicated and erroneous. Please correct the section headings. Section 3 is titled “Study on the reinforcement effect of MICP on unloading-damaged mudstone,” and Section 4 is identically titled. Additionally, Section 4.1 and 4.2 follow, but Section 5 is missing entirely (the manuscript jumps from Section 4.2 to Section 6 “Conclusions”). Please renumber sections sequentially (e.g., 3, 4, 5) and ensure no gaps or duplicates.

SEM analysis is critical for supporting the proposed micro-mechanisms. Without scale bars, the reader cannot evaluate the relative size of pores, cracks, or calcium carbonate precipitates. Please add scale bars to all SEM images and include the scale in the figure captions.

The definition of the unloading damage degree is not clear. In the text, the unloading damage degree is defined as 70%, 80%, and 90%, but no calculation formula is provided.

The statistical analysis of mechanical properties lacks measures of variability (e.g., standard deviation or confidence intervals), which is essential for interpreting the significance of the reported strength increases. The manuscript states that nine parallel specimens were prepared per condition (Section 2.3.1), but only single values are reported. Please include standard deviations or coefficients of variation for peak and residual strengths. This is particularly important given the natural heterogeneity of mudstone and the manual grouting process.

The manuscript would benefit from a brief discussion of the practical implications for field applications, especially regarding the injection of volcanic ash-assisted MICP into low-permeability mudstone. Mudstone is known for its low permeability. The laboratory study uses a pre-drilled central grout hole (6 mm diameter) to facilitate solution infiltration. In field conditions, achieving similar distribution of bacteria, cementation solution, and fine volcanic ash particles may be challenging. Please add a paragraph discussing potential field implementation challenges and possible solutions (e.g., grouting pressure, borehole spacing, or pre-conditioning).

Table 1 is not described clearly. Please redesign the table. It is suggested to use three groups of comparisons (unreinforced, MICP, MICP + volcanic ash), with each group including residual strength, peak strength and increase. Clearly present the complete data corresponding to all unloading damage degree.

Figure 4 is not clearly described. The two vertical axes (σ1 and σ3) overlap. It is recommended to separate them.

Figure 5, It is recommended to draw a clear schematic diagram to describe the process of microbial grouting reinforcement, rather than a blurry photograph.

Figure 12's mechanism diagram is too simplistic and does not correspond well to the description in Section 4.2.

Line 274, Fig. 13 is missing or the reference is incorrect.

There are numerous grammatical and spelling errors in the text. Please carefully review it.
