# Supplementary material for: Experimental study on the reinforcement performance of unloading-damaged mudstone by microbial grouting
Source: PLoS One. 2026 Aug 13;21(8):e0350062. doi: 10.1371/journal.pone.0350062 (PMC13472361; doi:10.1371/journal.pone.0350062)
Supplement: Supplementary file 2 [file pone.0350062.s003.docx]

**Response to the Editor’s Comments**

Thank you very much for your careful review and valuable comments on our manuscript. We have revised the manuscript thoroughly in accordance with your suggestions. All changes have been incorporated into the revised version, and our point-by-point responses are provided below.

**Reviewer #1 The authors carried out an experimental investigation on the reinforcement performance of unloading-damaged mudstone through microbial grouting. This paper holds a certain degree of research significance. Nevertheless, the innovation and research methods are mediocre. Consequently, I have pinpointed several aspects that necessitate revision prior to its potential final acceptance.**

**Comment 1:** Section numbering is duplicated and erroneous. Please correct the section headings. Section 3 is titled “Study on the reinforcement effect of MICP on unloading-damaged mudstone,” and Section 4 is identically titled. Additionally, Section 4.1 and 4.2 follow, but Section 5 is missing entirely (the manuscript jumps from Section 4.2 to Section 6 “Conclusions”). Please renumber sections sequentially (e.g., 3, 4, 5) and ensure no gaps or duplicates

**Response:** Thank you very much for the reviewer’s careful reading and valuable comments. In the revised manuscript, the relevant section titles and numbering have been corrected. Section 3 has been revised as the analysis of the swelling and mechanical properties of reinforced unloading-damaged mudstone, Section 4 has been revised as the analysis of microstructure and reinforcement mechanism, and the conclusions have been renumbered as Section 5, “Conclusions”. In addition, all section and subsection headings throughout the manuscript have been carefully checked to ensure that the numbering is sequential and that there are no missing sections, numbering skips, or duplicated titles.

**Comment 2:** SEM analysis is critical for supporting the proposed micro-mechanisms. Without scale bars, the reader cannot evaluate the relative size of pores, cracks, or calcium carbonate precipitates. Please add scale bars to all SEM images and include the scale in the figure captions.

**Response:** Thank you for the reviewer’s valuable comment. In the original manuscript, the SEM images did not include scale bars, which may have affected readers’ accurate understanding of the microscopic structural features and the spatial distribution scale of the mineralization products. In the revised manuscript, all SEM images have been reorganized, and corresponding scale bars have been added to each SEM image.

**Comment 3:** The definition of the unloading damage degree is not clear. In the text, the unloading damage degree is defined as 70%, 80%, and 90%, but no calculation formula is provided.

**Response:** Thank you for the reviewer’s careful reading and valuable comments. To address the unclear definition of the unloading damage degree in the original manuscript, additional explanations have been provided in Section 2.3 of the revised manuscript. It is clarified that the unloading damage degree in this study is characterized by the magnitude of confining pressure unloading. In essence, it represents a confining pressure unloading degree controlled by the stress path, rather than a value calculated from strain.

Meanwhile, the calculation formula for the unloading damage degree has been added to the main text:

$$\eta=\left[ \frac{\sigma_{3}^{0}-\sigma_{3}^{i}}{\sigma_{3}^{0}-\sigma_{3}^{f}} \right]\times100\%$$

**w**here σ₃⁰ is the initial confining pressure at the unloading point, $\sigma_{3}^{i}$ is the target confining pressure after unloading, and σ₃ᶠ is the confining pressure corresponding to specimen failure under the conventional unloading path. According to this equation, the target confining pressures of 2.6, 2.3, and 2.0 MPa used in this study correspond to the designed unloading damage degrees of 70%, 80%, and 90%, respectively. The relevant content has been supplemented in the revised manuscript to improve the clarity and reproducibility of the experimental methodology.

**Comment 4:** The statistical analysis of mechanical properties lacks measures of variability (e.g., standard deviation or confidence intervals), which is essential for interpreting the significance of the reported strength increases. The manuscript states that nine parallel specimens were prepared per condition (Section 2.3.1), but only single values are reported. Please include standard deviations or coefficients of variation for peak and residual strengths. This is particularly important given the natural heterogeneity of mudstone and the manual grouting process.

**Response:** Thank you for the reviewer’s valuable comment. The mechanical properties analysis in Section 3.2 has been supplemented and revised accordingly. The peak strength and residual strength of the parallel specimens in different groups were further statistically analyzed. A new statistical distribution figure has been added to the revised manuscript, and the coefficients of variation for the peak strength and residual strength of each group have been included in Table 1.

The results show that the strength data of different groups under various unloading damage degrees exhibit a certain degree of variability, which is related to the natural heterogeneity of mudstone and the spatial variation in mineralization product distribution during artificial grouting. Nevertheless, the overall distribution trend indicates that the peak strength and residual strength of the MICP group and the MICP-volcanic ash group are both higher than those of the unreinforced group, and the improvement is more pronounced in the MICP-volcanic ash group. This indicates that the reinforcement effect remains consistent even when the variability among specimens is considered. The relevant content has been supplemented in the corresponding section of the revised manuscript.

**Comment 5:** The manuscript would benefit from a brief discussion of the practical implications for field applications, especially regarding the injection of volcanic ash-assisted MICP into low-permeability mudstone. Mudstone is known for its low permeability. The laboratory study uses a pre-drilled central grout hole (6 mm diameter) to facilitate solution infiltration. In field conditions, achieving similar distribution of bacteria, cementation solution, and fine volcanic ash particles may be challenging. Please add a paragraph discussing potential field implementation challenges and possible solutions (e.g., grouting pressure, borehole spacing, or pre-conditioning).

**Response:** Thank you for the reviewer’s valuable comment. The practical implications and potential challenges associated with field application have been supplemented in the Discussion section of the revised manuscript. As correctly noted by the reviewer, intact mudstone generally has very low permeability. However, field investigation and laboratory observations indicate that, during subgrade excavation, the rock mass near the excavation surface gradually develops macro- and micro-cracks due to unloading damage. Water infiltration and deterioration along these cracks are among the main causes of subgrade distress. Therefore, the core objective of this study is to repair these macro- and micro-cracks through microbial grouting. The experimental results show that the microbial grout exhibits good diffusibility within these cracks.

For field application, the reinforcement effect can be further improved by reasonably arranging grouting boreholes, optimizing borehole spacing, adopting staged low-pressure cyclic grouting, and controlling the particle size of volcanic ash and the grout concentration, so as to enhance the uniform diffusion of bacterial suspension, cementation solution, and volcanic ash particles within the mudstone fracture network. In addition, field permeability tests and small-scale trial grouting should be conducted to optimize the grouting pressure, grouting volume, and number of grouting cycles. Excessively high grouting pressure should be avoided, as it may induce new crack propagation or local hydraulic splitting.

**Comment 6:** Table 1 is not described clearly. Please redesign the table. It is suggested to use three groups of comparisons (unreinforced, MICP, MICP + volcanic ash), with each group including residual strength, peak strength and increase. Clearly present the complete data corresponding to all unloading damage degree.

**Response:** Thank you for the reviewer’s valuable comment. Table 1 has been redesigned in the revised manuscript. In the original version, the data arrangement was not sufficiently intuitive, making it difficult to clearly compare the changes in mechanical properties under different reinforcement methods. In the revised version, Table 1 presents the residual strength, peak strength, and corresponding strength improvement rates of the unreinforced group, MICP group, and MICP-volcanic ash group under unloading damage degrees of 70%, 80%, and 90%, respectively. The improvement rates were calculated using the unreinforced group under the same unloading damage degree as the reference. In addition, the coefficients of variation of the residual strength and peak strength have been added to the table. The relevant content has been revised and supplemented in the corresponding sections of the manuscript.

**Comment 7:** Figure 4 is not clearly described. The two vertical axes (σ1 and σ3) overlap. It is recommended to separate them.

**Response:** Thank you for the reviewer’s careful review and constructive suggestion. In the original manuscript, the unloading stress path shown in Fig. 4 was not sufficiently clear, which could have led to confusion regarding the axial loading process and the confining pressure unloading process. In the revised version, the axial stress σ₁ and confining pressure σ₃ are plotted separately on the left and right vertical axes, respectively. Key parameters, including the initial axial stress, initial confining pressure, target confining pressure after unloading, and the confining pressure corresponding to unloading-induced failure, have also been clearly labeled.

In addition, the loading and unloading stages have been re-annotated, and the corresponding loading schematic has been retained to more clearly illustrate the experimental procedure. The relevant modification has been made in Fig. 4 of the revised manuscript.

**Comment 8:** Figure 5, It is recommended to draw a clear schematic diagram to describe the process of microbial grouting reinforcement, rather than a blurry photograph.

**Response:** Thank you for the reviewer’s valuable comment. In the original manuscript, Fig. 5 used a photograph of the experimental setup to illustrate the microbial grouting reinforcement process, which was not sufficiently clear. In the revised manuscript, Fig. 5 has been redesigned, and the original unclear photograph has been replaced with a clear schematic diagram.

The revised Fig. 5 schematically presents the main components and operational procedure of the microbial grouting reinforcement process, including the specimen, PVC mold, grouting hole, rubber plugs, injection paths of the bacterial suspension and cementation solution, the connection with the peristaltic pump, and the staged grouting procedure. The corresponding figure and related description have been replaced in the revised manuscript.

**Comment 9:** Figure 12's mechanism diagram is too simplistic and does not correspond well to the description in Section 4.2.

**Response:** Thank you for the reviewer’s valuable comment. In the original manuscript, the schematic diagram in Fig. 12 did not fully illustrate the reinforcement processes of MICP and MICP-volcanic ash treatments. Therefore, Fig. 12 has been redesigned and improved in the revised manuscript.

The revised Fig. 12 presents the microbial grouting reinforcement mechanism of damaged mudstone through two pathways: MICP reinforcement and MICP-volcanic ash reinforcement. For the MICP reinforcement process, the figure now includes key processes such as the entry and attachment of bacterial suspension into fractures, Ca²⁺ adsorption and calcium carbonate nucleation, mineralization deposition, and preferential precipitation at fracture constrictions and crack tips. These additions help explain the filling, sealing, and bridging effects of calcium carbonate precipitation in mudstone pores and fractures.

For the MICP-volcanic ash reinforcement process, the revised figure further illustrates the bacterial loading, protection, and retention effects of volcanic ash particles, as well as their initial filling effect, nucleation promotion, and possible composite cementation in fractures. These revisions more clearly demonstrate the enhancement effect of volcanic ash on MICP-induced mineralization and structural densification. The relevant modifications have been made in Section 4.2 of the revised manuscript.

**Comment 10:** Line 274, Fig. 13 is missing or the reference is incorrect.

**Response:** Thank you for the reviewer’s careful reading and correction. The figure number cited in this part was indeed inconsistent, which could have caused misunderstanding regarding the correspondence between the text and the figures. This figure reference has been corrected in the revised manuscript.

In addition, all figure numbers, figure captions, and in-text figure citations throughout the manuscript have been carefully checked to ensure consistency in the order of first appearance, numbering sequence, and citation positions. The relevant content has been revised accordingly in the manuscript.

**Comment 11:** There are numerous grammatical and spelling errors in the text. Please carefully review it.

**Response:** Thank you for the reviewer’s careful review and valuable comment. The original manuscript contained some grammatical errors, spelling mistakes, and imprecise expressions, which may have affected the readability and academic clarity of the paper. The entire manuscript has therefore been carefully checked and systematically polished.

In particular, we revised grammatical and spelling errors, corrected inappropriate punctuation, improved sentence fluency, and unified the use of technical terminology where inconsistencies existed. These revisions have improved the clarity, readability, and overall academic presentation of the manuscript.

**Reviewer #2:**

**Comment 1:** The authors prepared mudstone specimens with unloading degrees of 70%, 80%, and 90%, but failed to provide a clear definition or calculation formula for the "unloading degree." The text only mentions an initial confining pressure of 5 MPa, axial loading up to 80% of the peak strength (held constant), followed by unloading the confining pressure to target values of 2.0, 2.3, and 2.6 MPa. The correspondence between these target confining pressures and the stated unloading degrees (70%–90%) is not explained, nor is it specified whether the unloading degree is calculated based on stress or strain. Recommendation: Clearly define the unloading degree and provide the calculation process mapping each target confining pressure to its corresponding unloading degree. Additionally, supplement the manuscript with a confining pressure unloading path diagram and a detailed description of the stress path.

**Response:** Thank you for the reviewer’s valuable comment. In the original manuscript, the definition of the unloading degree, its calculation basis, and the correspondence between the target confining pressure and the unloading degree were not sufficiently clear. Therefore, the definition and calculation formula of the unloading degree have been supplemented in Section 2.3 of the revised manuscript.

In this study, the unloading degree is defined as a stress-based unloading index determined by the magnitude of confining pressure reduction, rather than a strain-based parameter. It is calculated as follows:

$$\eta=\left[ \frac{\sigma_{3}^{0}-\sigma_{3}^{i}}{\sigma_{3}^{0}-\sigma_{3}^{f}} \right]\times100\%$$

where$\eta$ is the unloading degree; $\sigma_{3}^{0}$ is the initial confining pressure at the unloading point, which is 5 MPa in this study; $\sigma_{3}^{i}$ is the target confining pressure after unloading; and $\sigma_{3}^{f}$ is the confining pressure corresponding to specimen failure under the conventional unloading path. According to the results of preliminary unloading failure tests, $\sigma_{3}^{f}$ was determined to be 1.6 MPa. Based on this equation, the target confining pressures of 2.6, 2.3, and 2.0 MPa correspond to unloading degrees of 70%, 80%, and 90%, respectively.

In addition, the confining pressure unloading path diagram has been supplemented and redrawn in the revised manuscript. Taking the 90% unloading process as an example, the revised figure clearly illustrates the variations in axial stress σ₁ and confining pressure σ₃ at different stages. The specific stress path has also been further clarified in the revised manuscript. First, the axial stress σ₁ and confining pressure σ₃ were synchronously increased to 5 MPa. Then, the confining pressure was kept constant while axial loading was continued. When the axial stress reached 80% of the peak strength under a confining pressure of 5 MPa, this point was defined as the unloading point. Subsequently, the axial load was kept constant, and the confining pressure was unloaded at a rate of 0.05 MPa/s to the target values of 2.6, 2.3, or 2.0 MPa, thereby preparing mudstone specimens with unloading degrees of 70%, 80%, and 90%, respectively.

Through these revisions, the definition, calculation procedure, and stress path of the unloading degree have been clearly explained.

**Comment 2:** The experimental design includes an unreinforced group, an MICP-only group, and an MICP + volcanic ash group; however, it lacks a "volcanic ash only (without MICP)" grouting group. Since volcanic ash itself possesses physical filling properties and potential pozzolanic activity, its independent effect may already contribute to the reinforcement of the mudstone. Without this control group, it is impossible to quantitatively evaluate the synergistic contributions of MICP and volcanic ash. Recommendation: Add a test group treated solely with volcanic ash grouting (using the same dosage and injection protocol) and subject it to identical mechanical and expansion tests. If supplementary experiments cannot be conducted, this limitation must be explicitly acknowledged in the discussion section, and the authors should avoid attributing all enhancement effects entirely to synergy.

**Response:** Thank you for the reviewer’s valuable comment. Due to limitations in specimen availability and the experimental schedule, a volcanic-ash-only grouting group has not been added in the present study. Following the reviewer’s suggestion, this limitation has been explicitly stated in the Discussion section of the revised manuscript, and the relevant descriptions have been carefully revised.

In the revised manuscript, the enhanced performance of the MICP-volcanic ash group is no longer entirely attributed to the synergistic effect between volcanic ash and MICP. Instead, it is described more cautiously as follows: the introduction of volcanic ash may further improve the structural integrity of mudstone through multiple mechanisms, including physical filling, provision of attachment sites for microorganisms, and promotion of calcium carbonate nucleation and deposition.

Meanwhile, it has been clearly stated that, due to the absence of a volcanic-ash-only treatment group, the relative contributions of the independent effect of volcanic ash and the combined effect of MICP-volcanic ash still need to be further quantified through controlled experiments in future studies.

**Comment 3:** The authors claim that nine parallel specimens were prepared for each test group, yet only typical stress-strain curves and mean strength values are presented, without standard deviations, coefficients of variation, or error bars. Given the inherent heterogeneity of natural mudstone and the variability associated with unloading damage, the lack of statistical evaluation severely undermines the reliability of the conclusions. It is recommended to refer to recent research papers in the introduction. Flow mechanism of grouting slurry in rough fracture based on CFD-DEM coupling method. Brittleness evaluation of gas-bearing coal based on statistical damage constitution model and energy evolution mechanism. Recommendation: Include standard deviations or confidence intervals in the text, and illustrate the scatter range of multiple tests using shaded bands or error bars in Figure 8. If the dataset is insufficient, please specify the actual number of samples used for statistical analysis (e.g., the count after excluding outliers).

**Response:** Thank you for pointing out this limitation. Both unloading damage and the artificial grouting process may introduce a certain degree of variability in the experimental results. Therefore, presenting only typical stress–strain curves and average strength values is insufficient to fully support the evaluation of the reinforcement effect. In the revised manuscript, the statistical analysis of the mechanical test results has been supplemented.

First, recent studies related to grout flow mechanisms in fractures, statistical damage models of geomaterials, and brittleness evaluation have been added to the Introduction to further improve the research background and clarify the necessity of statistical analysis. Second, based on the results of nine parallel specimens in each experimental group, the peak strength and residual strength were recalculated. The strength improvement rate and coefficient of variation were also added to Table 1 in the revised manuscript to quantitatively reflect the variability of the experimental results under different unloading damage degrees and reinforcement methods. In the statistical analysis, all nine parallel specimen results in each group were used, and no outliers were excluded.

Regarding the revision of Fig. 8, this figure is mainly used to present the typical stress–strain responses of specimens under different reinforcement methods during triaxial compression, including the compaction, elastic, plastic, post-peak softening, and residual stages. If multiple shaded bands or error bars were directly superimposed on Fig. 8, the curves would be easily obscured, which would affect the identification of the post-peak softening and residual strength stages. Therefore, to more clearly present the variability range of repeated tests, shaded bands were not directly added to Fig. 8. Instead, a new Fig. 9 has been added to show the statistical distributions of peak strength and residual strength for each group using box plots. This figure provides a more intuitive representation of the central tendency, variability, and distribution range of the data for different experimental groups. The relevant content has been revised and supplemented in the corresponding sections of the manuscript.

**Comment 4:** The authors qualitatively describe features such as "rough particle surfaces," "filled pores," and "localized generation of C-S-H gel" via SEM images, but no quantitative image analysis was performed (e.g., pore area fraction, calcium carbonate precipitation coverage rate, or particle contact length). Furthermore, neither EDS spectra nor XRD data were provided to confirm the presence of C-S-H. Recommendation: Supplement the study with quantitative porosity statistics derived from image processing; conduct EDS point scanning or elemental mapping to verify the presence of C-S-H and calcium carbonate; and include XRD phase analysis to clearly identify the mineralogical composition of the mineralization products.

**Response:** Thank you for the reviewer’s valuable comment. In the original manuscript, the discussion of pore filling, particle cementation, and local cementitious products was based only on qualitative observations from SEM images. The lack of quantitative image analysis and phase identification made it difficult to fully support the interpretation of the microscopic reinforcement mechanism. Therefore, the microstructural analysis in the revised manuscript has been supplemented and improved.

First, to address the insufficiency of quantitative SEM image analysis, surface porosity and fractal dimension analyses based on image processing have been added in the revised manuscript. By binarizing the SEM images of the unreinforced group, the MICP group, and the MICP-volcanic ash group, the changes in surface porosity under different treatment conditions were quantified, and the fractal dimensions of the pore structures were calculated. These parameters were used to quantitatively characterize the degree of microscopic pore filling and the changes in structural complexity. The results show that, after MICP reinforcement, the surface porosity decreased and the pore structure became denser. With the introduction of volcanic ash, the surface porosity decreased further, and the fractal dimension changed accordingly, indicating that volcanic ash-assisted MICP can further improve the pore-fracture structure of mudstone and the cementation state between particles.

Second, to address the insufficient identification of mineralization products, XRD phase analysis has been added in the revised manuscript. The XRD results show that the characteristic peaks related to calcium carbonate became more pronounced after reinforcement, indicating that the MICP process induced the formation of calcium carbonate mineralization products. In the MICP-volcanic ash group, the combined effect of the mineralization products and the original mineral components made the microstructure of the specimen denser. These results are consistent with the SEM observations and the quantitative image analysis results, further supporting the conclusion that MICP and MICP-volcanic ash improve the structural integrity of unloading-damaged mudstone by filling pores, sealing cracks, and enhancing interparticle cementation. The relevant revisions have been incorporated into Section 4.1 of the revised manuscript.

**Comment 5:** After each grouting cycle, the specimens were dried at 40°C for 12 hours. Since the optimal growth temperature for Bacillus cereus typically ranges from 30°C to 37°C, 40°C might inhibit or even kill some microorganisms, leading to reduced mineralization activity in subsequent cycles. The authors have not addressed the impact of this drying step on bacterial survival rates, nor have they verified whether urease activity is maintained at this temperature. Recommendation: Measure the changes in OD600 and urease activity of the bacterial suspension before and after drying, or alternatively, switch to air-drying at room temperature (25°C) with an extended drying duration. If 40°C is strictly necessary, supporting literature or experimental validation must be provided.

**Response:** Thank you for the reviewer’s professional comment. Drying at 40°C for 12 h may indeed have a certain influence on the survival rate and urease activity of residual bacteria within the pores of the specimens. The possible changes in microbial activity caused by this drying procedure were not sufficiently explained in the original manuscript, which was a limitation in the description of the experimental methodology.

In this study, drying at 40°C for 12 h was adopted to simulate the rapid moisture loss of the shallow layer of red-bed mudstone foundations after excavation under ventilation, sunlight exposure, and wetting–drying conditions. This treatment also helped promote the stable deposition of mineralization products in pores and fractures after each grouting cycle and provided a relatively consistent initial moisture condition for the subsequent cyclic grouting process. To reduce the influence of drying on the subsequent mineralization reaction, fresh bacterial suspension was reinjected in each grouting cycle. In addition, the OD600 value and urease activity of the bacterial suspension were measured before each grouting cycle, and the OD600 value of the injected bacterial suspension was controlled at approximately 1.5, thereby ensuring the consistency of microbial input conditions during the subsequent cyclic grouting process.

Accordingly, the engineering simulation purpose, experimental consideration, and possible influence of the 40°C drying treatment have been supplemented in Section 2.3 of the revised manuscript. In addition, the Discussion section further states that the survival rate and urease activity of residual bacteria within the specimen pores after drying at 40°C were not directly measured in this study. Therefore, the influence of this drying procedure on residual microbial activity remains a limitation of the present study. Future research will further investigate the effects of different drying temperatures and drying durations on bacterial survival, urease activity, calcium carbonate precipitation, and the resulting MICP reinforcement performance.
