# Supplementary material for: Experimental study on the reinforcement performance of unloading-damaged mudstone by microbial grouting
Source: PLoS One. 2026 Aug 13;21(8):e0350062. doi: 10.1371/journal.pone.0350062 (PMC13472361; doi:10.1371/journal.pone.0350062)
Supplement: Peer review history

# Peer review: Decision Letter 0

**DOI:** 10.1371/journal.pone.0350062.r001

**Authors:** Zhenhua Li

6 Jun 2026

Dear Dr. Zhu,

Thank you for submitting your manuscript to PLOS ONE. After careful consideration, we feel that it has merit but does not fully meet PLOS ONE’s publication criteria as it currently stands. Therefore, we invite you to submit a revised version of the manuscript that addresses the points raised during the review process.

You will see from the referees' comments that additional information needs to be provided, and we ask that this be provided, before we consider you manuscript further.

Please submit your revised manuscript by Aug 05 2026 11:59PM. If you will need more time than this to complete your revisions, please reply to this message or contact the journal office at plosone@plos.org. When you're ready to submit your revision, log on to https://www.editorialmanager.com/pone/ and select the 'Submissions Needing Revision' folder to locate your manuscript file.

- A letter that responds to each point raised by the academic editor and reviewer(s). You should upload this letter as a separate file labeled 'Response to Reviewers'.
- A marked-up copy of your manuscript that highlights changes made to the original version. You should upload this as a separate file labeled 'Revised Manuscript with Track Changes'.
- An unmarked version of your revised paper without tracked changes. You should upload this as a separate file labeled 'Manuscript'.

If you would like to make changes to your financial disclosure, please include your updated statement in your cover letter. Guidelines for resubmitting your figure files are available below the reviewer comments at the end of this letter.

If applicable, we recommend that you deposit your laboratory protocols in protocols.io to enhance the reproducibility of your results. Protocols.io assigns your protocol its own identifier (DOI) so that it can be cited independently in the future. For instructions see: https://journals.plos.org/plosone/s/submission-guidelines#loc-laboratory-protocols. Additionally, PLOS ONE offers an option for publishing peer-reviewed Lab Protocol articles, which describe protocols hosted on protocols.io. Read more information on sharing protocols at https://plos.org/protocols?utm_medium=editorial-email&utm_source=authorletters&utm_campaign=protocols.

As the corresponding author, your ORCID iD is verified in the submission system and will appear in the published article. PLOS supports the use of ORCID, and we encourage all coauthors to register for an ORCID iD and use it as well. Please encourage your coauthors to verify their ORCID iD within the submission system before final acceptance, as unverified ORCID iDs will not appear in the published article. *Only*  the individual author can complete the verification step; PLOS staff *cannot*  verify ORCID iDs on behalf of authors.

We look forward to receiving your revised manuscript.

Kind regards,

Zhenhua Li

Academic Editor

PLOS One

**Journal Requirements:**

When submitting your revision, we need you to address these additional requirements.

1. Please ensure that your manuscript meets PLOS ONE's style requirements, including those for file naming. The PLOS ONE style templates can be found at

https://journals.plos.org/plosone/s/file?id=wjVg/PLOSOne_formatting_sample_main_body.pdf and

https://journals.plos.org/plosone/s/file?id=ba62/PLOSOne_formatting_sample_title_authors_affiliations.pdf

2. We note that the grant information you provided in the ‘Funding Information’ and ‘Financial Disclosure’ sections do not match.

When you resubmit, please ensure that you provide the correct grant numbers for the awards you received for your study in the ‘Funding Information’ section.

3. Thank you for stating the following in the Acknowledgments Section of your manuscript:

“We thank the precious suggestions by anonymous reviewers and editors, which have greatly helped the improvement of the paper. This research was supported by the National Natural Science Foundation of China (NSFC) (Grant Nos. U22A20600、42507231), Natural Science Foundation Innovation Group Project of Hubei Province (Grant No. 2025AFA015), Talent Research Initiation Fund Program of China Three Gorges University (grant number 2024RCKJ021).”

We note that you have provided funding information that is not currently declared in your Funding Statement. However, funding information should not appear in the Acknowledgments section or other areas of your manuscript. We will only publish funding information present in the Funding Statement section of the online submission form.

Please remove any funding-related text from the manuscript and let us know how you would like to update your Funding Statement. Currently, your Funding Statement reads as follows:

“The author(s) received no specific funding for this work.”

Please include your amended statements within your cover letter; we will change the online submission form on your behalf.

4. PLOS requires an ORCID iD for the corresponding author in Editorial Manager on papers submitted after December 6th, 2016. Please ensure that you have an ORCID iD and that it is validated in Editorial Manager. To do this, go to ‘Update my Information’ (in the upper left-hand corner of the main menu), and click on the Fetch/Validate link next to the ORCID field. This will take you to the ORCID site and allow you to create a new iD or authenticate a pre-existing iD in Editorial Manager.

5. Thank you for stating the following in the Competing Interests section:

“The authors have declared that no competing interests exist.”

We note that one or more of the authors are employed by a commercial company:

- Power China Hubei Electric Engineering Co., Ltd.,
- Power China Jiangxi Hydropower Engineering Bureau Co., Ltd.

1. Please provide an amended Funding Statement declaring this commercial affiliation, as well as a statement regarding the Role of Funders in your study. If the funding organization did not play a role in the study design, data collection and analysis, decision to publish, or preparation of the manuscript and only provided financial support in the form of authors' salaries and/or research materials, please review your statements relating to the author contributions, and ensure you have specifically and accurately indicated the role(s) that these authors had in your study. You can update author roles in the Author Contributions section of the online submission form.

Please also include the following statement within your amended Funding Statement.

“The funder provided support in the form of salaries for authors [insert relevant initials], but did not have any additional role in the study design, data collection and analysis, decision to publish, or preparation of the manuscript. The specific roles of these authors are articulated in the ‘author contributions’ section.”

If your commercial affiliation did play a role in your study, please state and explain this role within your updated Funding Statement.

2. Please also provide an updated Competing Interests Statement declaring this commercial affiliation along with any other relevant declarations relating to employment, consultancy, patents, products in development, or marketed products, etc.

Within your Competing Interests Statement, please confirm that this commercial affiliation does not alter your adherence to all PLOS ONE policies on sharing data and materials by including the following statement: "This does not alter our adherence to  PLOS ONE policies on sharing data and materials.” (as detailed online in our guide for authors http://journals.plos.org/plosone/s/competing-interests) . If this adherence statement is not accurate and  there are restrictions on sharing of data and/or materials, please state these. Please note that we cannot proceed with consideration of your article until this information has been declared.

Please include both an updated Funding Statement and Competing Interests Statement in your cover letter. We will change the online submission form on your behalf.

6. We note that Figures 1, 3,5, and 6  in your submission contain copyrighted images. All PLOS content is published under the Creative Commons Attribution License (CC BY 4.0), which means that the manuscript, images, and Supporting Information files will be freely available online, and any third party is permitted to access, download, copy, distribute, and use these materials in any way, even commercially, with proper attribution. For more information, see our copyright guidelines: http://journals.plos.org/plosone/s/licenses-and-copyright.

We require you to either (1) present written permission from the copyright holder to publish these figures specifically under the CC BY 4.0 license, or (2) remove the figures from your submission:

1. You may seek permission from the original copyright holder of Figure(s) [#] to publish the content specifically under the CC BY 4.0 license.

We recommend that you contact the original copyright holder with the Content Permission Form (http://journals.plos.org/plosone/s/file?id=7c09/content-permission-form.pdf) and the following text:

“I request permission for the open-access journal PLOS ONE to publish XXX under the Creative Commons Attribution License (CCAL) CC BY 4.0 (http://creativecommons.org/licenses/by/4.0/). Please be aware that this license allows unrestricted use and distribution, even commercially, by third parties. Please reply and provide explicit written permission to publish XXX under a CC BY license and complete the attached form.”

Please upload the completed Content Permission Form or other proof of granted permissions as an "Other" file with your submission.

In the figure caption of the copyrighted figure, please include the following text: “Reprinted from [ref] under a CC BY license, with permission from [name of publisher], original copyright [original copyright year].”

2. If you are unable to obtain permission from the original copyright holder to publish these figures under the CC BY 4.0 license or if the copyright holder’s requirements are incompatible with the CC BY 4.0 license, please either i) remove the figure or ii) supply a replacement figure that complies with the CC BY 4.0 license. Please check copyright information on all replacement figures and update the figure caption with source information. If applicable, please specify in the figure caption text when a figure is similar but not identical to the original image and is therefore for illustrative purposes only.

7. If the reviewer comments include a recommendation to cite specific previously published works, please review and evaluate these publications to determine whether they are relevant and should be cited. There is no requirement to cite these works unless the editor has indicated otherwise.

**Additional Editor Comments:**

You will see from the referees' comments that additional information needs to be provided, and we ask that this be provided, before we consider you manuscript further.

[Note: HTML markup is below. Please do not edit.]

Reviewers' comments:

Reviewer's Responses to Questions

**Comments to the Author**

1. Is the manuscript technically sound, and do the data support the conclusions?

Reviewer #1: Partly

Reviewer #2: Yes

**********

2. Has the statistical analysis been performed appropriately and rigorously?

Reviewer #1: No

Reviewer #2: Yes

**********

3. Have the authors made all data underlying the findings in their manuscript fully available?

The PLOS Data policy

Reviewer #1: Yes

Reviewer #2: Yes

**********

4. Is the manuscript presented in an intelligible fashion and written in standard English?

Reviewer #1: No

Reviewer #2: Yes

**********

Reviewer #1: Comments to the Authors,

The authors carried out an experimental investigation on the reinforcement performance of unloading-damaged mudstone through microbial grouting. This paper holds a certain degree of research significance. Nevertheless, the innovation and research methods are mediocre. Consequently, I have pinpointed several aspects that necessitate revision prior to its potential final acceptance.

specific comments�

Section numbering is duplicated and erroneous. Please correct the section headings. Section 3 is titled “Study on the reinforcement effect of MICP on unloading-damaged mudstone,” and Section 4 is identically titled. Additionally, Section 4.1 and 4.2 follow, but Section 5 is missing entirely (the manuscript jumps from Section 4.2 to Section 6 “Conclusions”). Please renumber sections sequentially (e.g., 3, 4, 5) and ensure no gaps or duplicates.

SEM analysis is critical for supporting the proposed micro-mechanisms. Without scale bars, the reader cannot evaluate the relative size of pores, cracks, or calcium carbonate precipitates. Please add scale bars to all SEM images and include the scale in the figure captions.

The definition of the unloading damage degree is not clear. In the text, the unloading damage degree is defined as 70%, 80%, and 90%, but no calculation formula is provided.

The statistical analysis of mechanical properties lacks measures of variability (e.g., standard deviation or confidence intervals), which is essential for interpreting the significance of the reported strength increases. The manuscript states that nine parallel specimens were prepared per condition (Section 2.3.1), but only single values are reported. Please include standard deviations or coefficients of variation for peak and residual strengths. This is particularly important given the natural heterogeneity of mudstone and the manual grouting process.

The manuscript would benefit from a brief discussion of the practical implications for field applications, especially regarding the injection of volcanic ash-assisted MICP into low-permeability mudstone. Mudstone is known for its low permeability. The laboratory study uses a pre-drilled central grout hole (6 mm diameter) to facilitate solution infiltration. In field conditions, achieving similar distribution of bacteria, cementation solution, and fine volcanic ash particles may be challenging. Please add a paragraph discussing potential field implementation challenges and possible solutions (e.g., grouting pressure, borehole spacing, or pre-conditioning).

Table 1 is not described clearly. Please redesign the table. It is suggested to use three groups of comparisons (unreinforced, MICP, MICP + volcanic ash), with each group including residual strength, peak strength and increase. Clearly present the complete data corresponding to all unloading damage degree.

Figure 4 is not clearly described. The two vertical axes (σ1 and σ3) overlap. It is recommended to separate them.

Figure 5, It is recommended to draw a clear schematic diagram to describe the process of microbial grouting reinforcement, rather than a blurry photograph.

Figure 12's mechanism diagram is too simplistic and does not correspond well to the description in Section 4.2.

Line 274, Fig. 13 is missing or the reference is incorrect.

There are numerous grammatical and spelling errors in the text. Please carefully review it.

Reviewer #2: 1.The authors prepared mudstone specimens with unloading degrees of 70%, 80%, and 90%, but failed to provide a clear definition or calculation formula for the "unloading degree." The text only mentions an initial confining pressure of 5 MPa, axial loading up to 80% of the peak strength (held constant), followed by unloading the confining pressure to target values of 2.0, 2.3, and 2.6 MPa. The correspondence between these target confining pressures and the stated unloading degrees (70%–90%) is not explained, nor is it specified whether the unloading degree is calculated based on stress or strain. Recommendation: Clearly define the unloading degree and provide the calculation process mapping each target confining pressure to its corresponding unloading degree. Additionally, supplement the manuscript with a confining pressure unloading path diagram and a detailed description of the stress path.

2.The experimental design includes an unreinforced group, an MICP-only group, and an MICP + volcanic ash group; however, it lacks a "volcanic ash only (without MICP)" grouting group. Since volcanic ash itself possesses physical filling properties and potential pozzolanic activity, its independent effect may already contribute to the reinforcement of the mudstone. Without this control group, it is impossible to quantitatively evaluate the synergistic contributions of MICP and volcanic ash. Recommendation: Add a test group treated solely with volcanic ash grouting (using the same dosage and injection protocol) and subject it to identical mechanical and expansion tests. If supplementary experiments cannot be conducted, this limitation must be explicitly acknowledged in the discussion section, and the authors should avoid attributing all enhancement effects entirely to synergy.

3.The authors claim that nine parallel specimens were prepared for each test group, yet only typical stress-strain curves and mean strength values are presented, without standard deviations, coefficients of variation, or error bars. Given the inherent heterogeneity of natural mudstone and the variability associated with unloading damage, the lack of statistical evaluation severely undermines the reliability of the conclusions. It is recommended to refer to recent research papers in the introduction. Flow mechanism of grouting slurry in rough fracture based on CFD-DEM coupling method. Brittleness evaluation of gas-bearing coal based on statistical damage constitution model and energy evolution mechanism. Recommendation: Include standard deviations or confidence intervals in the text, and illustrate the scatter range of multiple tests using shaded bands or error bars in Figure 8. If the dataset is insufficient, please specify the actual number of samples used for statistical analysis (e.g., the count after excluding outliers).

4.The authors qualitatively describe features such as "rough particle surfaces," "filled pores," and "localized generation of C-S-H gel" via SEM images, but no quantitative image analysis was performed (e.g., pore area fraction, calcium carbonate precipitation coverage rate, or particle contact length). Furthermore, neither EDS spectra nor XRD data were provided to confirm the presence of C-S-H. Recommendation: Supplement the study with quantitative porosity statistics derived from image processing; conduct EDS point scanning or elemental mapping to verify the presence of C-S-H and calcium carbonate; and include XRD phase analysis to clearly identify the mineralogical composition of the mineralization products.

5.After each grouting cycle, the specimens were dried at 40°C for 12 hours. Since the optimal growth temperature for Bacillus cereus typically ranges from 30°C to 37°C, 40°C might inhibit or even kill some microorganisms, leading to reduced mineralization activity in subsequent cycles. The authors have not addressed the impact of this drying step on bacterial survival rates, nor have they verified whether urease activity is maintained at this temperature. Recommendation: Measure the changes in OD600 and urease activity of the bacterial suspension before and after drying, or alternatively, switch to air-drying at room temperature (25°C) with an extended drying duration. If 40°C is strictly necessary, supporting literature or experimental validation must be provided.

**********

what does this mean?). If published, this will include your full peer review and any attached files.

If you choose “no”, your identity will remain anonymous but your review may still be made public.

**Do you want your identity to be public for this peer review?**  For information about this choice, including consent withdrawal, please see our Privacy Policy

Reviewer #1: No

Reviewer #2: No

**********

[NOTE: If reviewer comments were submitted as an attachment file, they will be attached to this email and accessible via the submission site. Please log into your account, locate the manuscript record, and check for the action link "View Attachments". If this link does not appear, there are no attachment files.]

To ensure your figures meet our technical requirements, please review our figure guidelines: https://journals.plos.org/plosone/s/figures

You may also use PLOS’s free figure tool, NAAS, to help you prepare publication quality figures: https://journals.plos.org/plosone/s/figures#loc-tools-for-figure-preparation.

NAAS will assess whether your figures meet our technical requirements by comparing each figure against our figure specifications.

Attachment Submitted filename: Comments.docx

# Author comment: Author response to Decision Letter 1

**DOI:** 10.1371/journal.pone.0350062.r002

1 Jul 2026

Response to the Editor’s Comments

Thank you very much for your careful review and valuable comments on our manuscript. We have revised the manuscript thoroughly in accordance with your suggestions. All changes have been incorporated into the revised version, and our point-by-point responses are provided below.

Reviewer #1 The authors carried out an experimental investigation on the reinforcement performance of unloading-damaged mudstone through microbial grouting. This paper holds a certain degree of research significance. Nevertheless, the innovation and research methods are mediocre. Consequently, I have pinpointed several aspects that necessitate revision prior to its potential final acceptance.

Comment 1: Section numbering is duplicated and erroneous. Please correct the section headings. Section 3 is titled “Study on the reinforcement effect of MICP on unloading-damaged mudstone,” and Section 4 is identically titled. Additionally, Section 4.1 and 4.2 follow, but Section 5 is missing entirely (the manuscript jumps from Section 4.2 to Section 6 “Conclusions”). Please renumber sections sequentially (e.g., 3, 4, 5) and ensure no gaps or duplicates

Response: Thank you very much for the reviewer’s careful reading and valuable comments. In the revised manuscript, the relevant section titles and numbering have been corrected. Section 3 has been revised as the analysis of the swelling and mechanical properties of reinforced unloading-damaged mudstone, Section 4 has been revised as the analysis of microstructure and reinforcement mechanism, and the conclusions have been renumbered as Section 5, “Conclusions”. In addition, all section and subsection headings throughout the manuscript have been carefully checked to ensure that the numbering is sequential and that there are no missing sections, numbering skips, or duplicated titles.

Comment 2: SEM analysis is critical for supporting the proposed micro-mechanisms. Without scale bars, the reader cannot evaluate the relative size of pores, cracks, or calcium carbonate precipitates. Please add scale bars to all SEM images and include the scale in the figure captions.

Response: Thank you for the reviewer’s valuable comment. In the original manuscript, the SEM images did not include scale bars, which may have affected readers’ accurate understanding of the microscopic structural features and the spatial distribution scale of the mineralization products. In the revised manuscript, all SEM images have been reorganized, and corresponding scale bars have been added to each SEM image.

Comment 3: The definition of the unloading damage degree is not clear. In the text, the unloading damage degree is defined as 70%, 80%, and 90%, but no calculation formula is provided.

Response: Thank you for the reviewer’s careful reading and valuable comments. To address the unclear definition of the unloading damage degree in the original manuscript, additional explanations have been provided in Section 2.3 of the revised manuscript. It is clarified that the unloading damage degree in this study is characterized by the magnitude of confining pressure unloading. In essence, it represents a confining pressure unloading degree controlled by the stress path, rather than a value calculated from strain.

Meanwhile, the calculation formula for the unloading damage degree has been added to the main text:

η=σ30−σ3iσ30−σ3f×100%

where σ₃⁰ is the initial confining pressure at the unloading point, σ3i is the target confining pressure after unloading, and σ₃ᶠ is the confining pressure corresponding to specimen failure under the conventional unloading path. According to this equation, the target confining pressures of 2.6, 2.3, and 2.0 MPa used in this study correspond to the designed unloading damage degrees of 70%, 80%, and 90%, respectively. The relevant content has been supplemented in the revised manuscript to improve the clarity and reproducibility of the experimental methodology.

Comment 4: The statistical analysis of mechanical properties lacks measures of variability (e.g., standard deviation or confidence intervals), which is essential for interpreting the significance of the reported strength increases. The manuscript states that nine parallel specimens were prepared per condition (Section 2.3.1), but only single values are reported. Please include standard deviations or coefficients of variation for peak and residual strengths. This is particularly important given the natural heterogeneity of mudstone and the manual grouting process.

Response: Thank you for the reviewer’s valuable comment. The mechanical properties analysis in Section 3.2 has been supplemented and revised accordingly. The peak strength and residual strength of the parallel specimens in different groups were further statistically analyzed. A new statistical distribution figure has been added to the revised manuscript, and the coefficients of variation for the peak strength and residual strength of each group have been included in Table 1.

The results show that the strength data of different groups under various unloading damage degrees exhibit a certain degree of variability, which is related to the natural heterogeneity of mudstone and the spatial variation in mineralization product distribution during artificial grouting. Nevertheless, the overall distribution trend indicates that the peak strength and residual strength of the MICP group and the MICP-volcanic ash group are both higher than those of the unreinforced group, and the improvement is more pronounced in the MICP-volcanic ash group. This indicates that the reinforcement effect remains consistent even when the variability among specimens is considered. The relevant content has been supplemented in the corresponding section of the revised manuscript.

Comment 5: The manuscript would benefit from a brief discussion of the practical implications for field applications, especially regarding the injection of volcanic ash-assisted MICP into low-permeability mudstone. Mudstone is known for its low permeability. The laboratory study uses a pre-drilled central grout hole (6 mm diameter) to facilitate solution infiltration. In field conditions, achieving similar distribution of bacteria, cementation solution, and fine volcanic ash particles may be challenging. Please add a paragraph discussing potential field implementation challenges and possible solutions (e.g., grouting pressure, borehole spacing, or pre-conditioning).

Response: Thank you for the reviewer’s valuable comment. The practical implications and potential challenges associated with field application have been supplemented in the Discussion section of the revised manuscript. As correctly noted by the reviewer, intact mudstone generally has very low permeability. However, field investigation and laboratory observations indicate that, during subgrade excavation, the rock mass near the excavation surface gradually develops macro- and micro-cracks due to unloading damage. Water infiltration and deterioration along these cracks are among the main causes of subgrade distress. Therefore, the core objective of this study is to repair these macro- and micro-cracks through microbial grouting. The experimental results show that the microbial grout exhibits good diffusibility within these cracks.

For field application, the reinforcement effect can be further improved by reasonably arranging grouting boreholes, optimizing borehole spacing, adopting staged low-pressure cyclic grouting, and controlling the particle size of volcanic ash and the grout concentration, so as to enhance the uniform diffusion of bacterial suspension, cementation solution, and volcanic ash particles within the mudstone fracture network. In addition, field permeability tests and small-scale trial grouting should be conducted to optimize the grouting pressure, grouting volume, and number of grouting cycles. Excessively high grouting pressure should be avoided, as it may induce new crack propagation or local hydraulic splitting.

Comment 6: Table 1 is not described clearly. Please redesign the table. It is suggested to use three groups of comparisons (unreinforced, MICP, MICP + volcanic ash), with each group including residual strength, peak strength and increase. Clearly present the complete data corresponding to all unloading damage degree.

Response: Thank you for the reviewer’s valuable comment. Table 1 has been redesigned in the revised manuscript. In the original version, the data arrangement was not sufficiently intuitive, making it difficult to clearly compare the changes in mechanical properties under different reinforcement methods. In the revised version, Table 1 presents the residual strength, peak strength, and corresponding strength improvement rates of the unreinforced group, MICP group, and MICP-volcanic ash group under unloading damage degrees of 70%, 80%, and 90%, respectively. The improvement rates were calculated using the unreinforced group under the same unloading damage degree as the reference. In addition, the coefficients of variation of the residual strength and peak strength have been added to the table. The relevant content has been revised and supplemented in the corresponding sections of the manuscript.

Comment 7: Figure 4 is not clearly described. The two vertical axes (σ1 and σ3) overlap. It is recommended to separate them.

Response: Thank you for the reviewer’s careful review and constructive suggestion. In the original manuscript, the unloading stress path shown in Fig. 4 was not sufficiently clear, which could have led to confusion regarding the axial loading process and the confining pressure unloading process. In the revised version, the axial stress σ₁ and confining pressure σ₃ are plotted separately on the left and right vertical axes, respectively. Key parameters, including the initial axial stress, initial confining pressure, target confining pressure after unloading, and the confining pressure corresponding to unloading-induced failure, have also been clearly labeled.

In addition, the loading and unloading stages have been re-annotated, and the corresponding loading schematic has been retained to more clearly illustrate the experimental procedure. The relevant modification has been made in Fig. 4 of the revised manuscript.

Comment 8: Figure 5, It is recommended to draw a clear schematic diagram to describe the process of microbial grouting reinforcement, rather than a blurry photograph.

Response: Thank you for the reviewer’s valuable comment. In the original manuscript, Fig. 5 used a photograph of the experimental setup to illustrate the microbial grouting reinforcement process, which was not sufficiently clear. In the revised manuscript, Fig. 5 has been redesigned, and the original unclear photograph has been replaced with a clear schematic diagram.

The revised Fig. 5 schematically presents the main components and operational procedure of the microbial grouting reinforcement process, including the specimen, PVC mold, grouting hole, rubber plugs, injection paths of the bacterial suspension and cementation solution, the connection with the peristaltic pump, and the staged grouting procedure. The corresponding figure and related description have been replaced in the revised manuscript.

Comment 9: Figure 12's mechanism diagram is too simplistic and does not correspond well to the description in Section 4.2.

Response: Thank you for the reviewer’s valuable comment. In the original manuscript, the schematic diagram in Fig. 12 did not fully illustrate the reinforcement processes of MICP and MICP-volcanic ash treatments. Therefore, Fig. 12 has been redesigned and improved in the revised manuscript.

The revised Fig. 12 presents the microbial grouting reinforcement mechanism of damaged mudstone through two pathways: MICP reinforcement and MICP-volcanic ash reinforcement. For the MICP reinforcement process, the figure now includes key processes such as the entry and attachment of bacterial suspension into fractures, Ca²⁺ adsorption and calcium carbonate nucleation, mineralization deposition, and preferential precipitation at fracture constrictions and crack tips. These additions help explain the filling, sealing, and bridging effects of calcium carbonate precipitation in mudstone pores and fractures.

For the MICP-volcanic ash reinforcement process, the revised figure further illustrates the bacterial loading, protection, and retention effects of volcanic ash particles, as well as their initial filling effect, nucleation promotion, and possible composite cementation in fractures. These revisions more clearly demonstrate the enhancement effect of volcanic ash on MICP-induced mineralization and structural densification. The relevant modifications have been made in Section 4.2 of the revised manuscript.

Comment 10: Line 274, Fig. 13 is missing or the reference is incorrect.

Response: Thank you for the reviewer’s careful reading and correction. The figure number cited in this part was indeed inconsistent, which could have caused misunderstanding regarding the correspondence between the text and the figures. This figure reference has been corrected in the revised manuscript.

In addition, all figure numbers, figure captions, and in-text figure citations throughout the manuscript have been carefully checked to ensure consistency in the order of first appearance, numbering sequence, and citation positions. The relevant content has been revised accordingly in the manuscript.

Comment 11: There are numerous grammatical and spelling errors in the text. Please carefully review it.

Response: Thank you for the reviewer’s careful review and valuable comment. The original manuscript contained some grammatical errors, spelling mistakes, and imprecise expressions, which may have affected the readability and academic clarity of the paper. The entire manuscript has therefore been carefully checked and systematically polished.

In particular, we revised grammatical and spelling errors, corrected inappropriate punctuation, improved sentence fluency, and unified the use of technical terminology where inconsistencies existed. These revisions have improved the clarity, readability, and overall academic presentation of the manuscript.

Reviewer #2:

Comment 1: The authors prepared mudstone specimens with unloading degrees of 70%, 80%, and 90%, but failed to provide a clear definition or calculation formula for the "unloading degree." The text only mentions an initial confining pressure of 5 MPa, axial loading up to 80% of the peak strength (held constant), followed by unloading the confining pressure to target values of 2.0, 2.3, and 2.6 MPa. The correspondence between these target confining pressures and the stated unloading degrees (70%–90%) is not explained, nor is it specified whether the unloading degree is calculated based on stress or strain. Recommendation: Clearly define the unloading degree and provide the calculation process mapping each target confining pressure to its corresponding unloading degree. Additionally, supplement the manuscript with a confining pressure unloading path diagram and a detailed description of the stress path.

Response: Thank you for the reviewer’s valuable comment. In the original manuscript, the definition of the unloading degree, its calculation basis, and the correspondence between the target confining pressure and the unloading degree were not sufficiently clear. Therefore, the definition and calculation formula of the unloading degree have been supplemented in Section 2.3 of the revised manuscript.

In this study, the unloading degree is defined as a stress-based unloading index determined by the magnitude of confining pressure reduction, rather than a strain-based parameter. It is calculated as follows:

η=σ30−σ3iσ30−σ3f×100%

whereη is the unloading degree; σ30 is the initial confining pressure at the unloading point, which is 5 MPa in this study; σ3i is the target confining pressure after unloading; and σ3f is the confining pressure corresponding to specimen failure under the conventional unloading path. According to the results of preliminary unloading failure tests, σ3f was determined to be 1.6 MPa. Based on this equation, the target confining pressures of 2.6, 2.3, and 2.0 MPa correspond to unloading degrees of 70

Attachment Submitted filename: renamed_8a15c.docx

# Peer review: Decision Letter 1

**DOI:** 10.1371/journal.pone.0350062.r003

**Authors:** Zhenhua Li

19 Jul 2026

Experimental Study on the Reinforcement Performance of Unloading-Damaged Mudstone by Microbial Grouting

PONE-D-26-22352R1

Dear Dr. Zhu,

We’re pleased to inform you that your manuscript has been judged scientifically suitable for publication and will be formally accepted for publication once it meets all outstanding technical requirements.

Within one week, you’ll receive an e-mail detailing the required amendments. When these have been addressed, you’ll receive a formal acceptance letter and your manuscript will be scheduled for publication.

An invoice will be generated when your article is formally accepted. Please note, if your institution has a publishing partnership with PLOS and your article meets the relevant criteria, all or part of your publication costs will be covered. Please make sure your user information is up-to-date by logging into Editorial Manager at Editorial Manager® and clicking the ‘Update My Information' link at the top of the page. For questions related to billing, please contact billing support.

If your institution or institutions have a press office, please notify them about your upcoming paper to help maximize its impact. If they’ll be preparing press materials, please inform our press team as soon as possible -- no later than 48 hours after receiving the formal acceptance. Your manuscript will remain under strict press embargo until 2 pm Eastern Time on the date of publication. For more information, please contact onepress@plos.org.

Kind regards,

Zhenhua Li

Academic Editor

PLOS One

Additional Editor Comments (optional):

accept

Reviewers' comments:

Reviewer's Responses to Questions

**Comments to the Author**

Reviewer #1: All comments have been addressed

Reviewer #2: All comments have been addressed

**********

2. Is the manuscript technically sound, and do the data support the conclusions?

Reviewer #1: (No Response)

Reviewer #2: Yes

**********

3. Has the statistical analysis been performed appropriately and rigorously?

Reviewer #1: (No Response)

Reviewer #2: Yes

**********

4. Have the authors made all data underlying the findings in their manuscript fully available?

The PLOS Data policy

Reviewer #1: (No Response)

Reviewer #2: Yes

**********

5. Is the manuscript presented in an intelligible fashion and written in standard English?

Reviewer #1: (No Response)

Reviewer #2: Yes

**********

Reviewer #1: (No Response)

Reviewer #2: accept.

To address the structural deterioration and water-induced swelling of mudstone caused by unloading damage during foundation excavation, MICP and MICP-volcanic ash grouting reinforcement tests were conducted.

**********

what does this mean?). If published, this will include your full peer review and any attached files.

If you choose “no”, your identity will remain anonymous but your review may still be made public.

**Do you want your identity to be public for this peer review?**  For information about this choice, including consent withdrawal, please see our Privacy Policy

Reviewer #1: No

Reviewer #2: No

**********

# Editor report: Acceptance letter

**DOI:** 10.1371/journal.pone.0350062.r004

**Authors:** Zhenhua Li

PONE-D-26-22352R1

PLOS One

Dear Dr. Zhu,

I'm pleased to inform you that your manuscript has been deemed suitable for publication in PLOS One. Congratulations! Your manuscript is now being handed over to our production team.

At this stage, our production department will prepare your paper for publication. This includes ensuring the following:

* All references, tables, and figures are properly cited

* All relevant supporting information is included in the manuscript submission,

* There are no issues that prevent the paper from being properly typeset

You will receive further instructions from the production team, including instructions on how to review your proof when it is ready. Please keep in mind that we are working through a large volume of accepted articles, so please give us a few days to review your paper and let you know the next and final steps.

Lastly, if your institution or institutions have a press office, please let them know about your upcoming paper now to help maximize its impact. If they'll be preparing press materials, please inform our press team within the next 48 hours. Your manuscript will remain under strict press embargo until 2 pm Eastern Time on the date of publication. For more information, please contact onepress@plos.org.

You will receive an invoice from PLOS for your publication fee after your manuscript has reached the completed accept phase. If you receive an email requesting payment before acceptance or for any other service, this may be a phishing scheme. Learn how to identify phishing emails and protect your accounts at https://explore.plos.org/phishing.

If we can help with anything else, please email us at customercare@plos.org.

Thank you for submitting your work to PLOS ONE and supporting open access.

Kind regards,

PLOS ONE Editorial Office Staff

on behalf of

Professor Zhenhua Li

Academic Editor

PLOS One
